# Time-lagged recurrence: A data-driven method to estimate the predictability of dynamical systems

**DOI:** 10.1073/pnas.2420252122

**Published:** 2025-05-16

**Authors:** Chenyu Dong, Davide Faranda, Adriano Gualandi, Valerio Lucarini, Gianmarco Mengaldo

**Affiliations:** ^a^Department of Mechanical Engineering, National University of Singapore, Singapore 117575, Singapore; ^b^Laboratoire des Sciences du Climat et de l’Environnement, Commissariat á l’énergie atomique et aux énergies alternatives Saclay l’Orme des Merisiers Unité Mixte de Recherche 8212 CEA-Centre National de la Recherche Scientifique-Université de Versailles - Saint-Quentin-en-Yvelines, Université Paris-Saclay & Institut Pierre Simon Laplace, 91191, Gif-sur-Yvette, France; ^c^London Mathematical Laboratory, London W6 8RH, United Kingdom; ^d^Laboratoire de Météorologie Dynamique/IPSL, École Normale Supérieure, Paris Sciences et Lettres Research University, Sorbonne Université, École Polytechnique, Paris, CNRS, Paris 75005, France; ^e^Bullard Laboratories, Department of Earth Sciences, University of Cambridge, Cambridge CB2 3EQ, United Kingdom; ^f^Osservatorio Nazionale Terremoti, Istituto Nazionale di Geofisica e Vulcanologia, Rome 00143, Italy; ^g^School of Computing and Mathematical Sciences, University of Leicester, Leicester, LE1 7RH, United Kingdom; ^h^Department of Mathematics, National University of Singapore, Singapore 119076, Singapore

**Keywords:** predictability, dynamical systems, data-driven methods, local dynamical indices

## Abstract

Nonlinear dynamical systems are ubiquitous in nature and they are hard to forecast. Not only they may be sensitive to small perturbations in their initial conditions, but they are often composed of processes acting at multiple scales. Classical approaches based on the Lyapunov spectrum rely on the knowledge of the dynamic forward operator, or of a data-derived approximation of it. This operator is typically unknown, or the data are too noisy to derive its faithful representation. Here, we propose a data-driven approach to analyze the local predictability of dynamical systems. This method, based on the concept of recurrence, is closely linked to the well-established framework of local dynamical indices. When applied to both idealized systems and real-world datasets arising from large-scale atmospheric fields, our approach proves its effectiveness in estimating local predictability. Additionally, we discuss its relationship with other local dynamical indices, and how it reveals the scale-dependent nature of predictability. Furthermore, we explore its link to information theory, its extension that includes a weighting strategy, and its real-time application. We believe these aspects collectively demonstrate its potential as a powerful diagnostic tool for complex systems.

## Significance

In many complex systems, predictability can be substantially state-dependent. We propose here a purely data-driven approach for estimating the local predictability at different time scales. The effectiveness of our approach is validated against existing knowledge, and its relationship to commonly used metrics in the dynamical systems literature is discussed. Our approach provides useful and convincing insights for both simple and real-world systems, including large-scale atmospheric circulation patterns in the Euro-Atlantic sector, clarifying key aspects of their predictability. Additionally, it can be used as a diagnostic tool in existing data-driven workflows to better understand the behavior of dynamical systems, opening a wide range of research opportunities.

Systems that evolve in time according to specific rules are known as dynamical systems, and the associated field of study is called dynamical system theory. There is a rich history on this topic, starting from the 1600s, when Newton developed calculus and classical mechanics (1), to the 1800s, when Poincaré developed geometrical approaches to the study of dynamical systems (2). In the 1900s, the field flourished, with the pioneering contributions of Birkhoff, Kolmogorov, Moser, Arnold, Lorenz, Ruelle, Takens, May, and Feigenbaum, among others, who laid the foundations of modern dynamical system theory, including works on oscillators, chaos, and fractals, well summarized by Strogatz (3).

Many real-world systems are made of multiple interacting parts, giving rise to complex dynamical systems. A paradigm for describing such systems relies on considering high-dimensional deterministic models, which are often characterized by chaotic behavior and multiscale dynamics (4, 5). A separate paradigm relies on considering stochastic dynamics, where randomness is explicitly introduced in the system (6, 7). When constructing reduced order models of high dimensional systems, stochastic dynamics is an emergent property. This is at the core of the Hasselmann’s program for climate science (8, 9) and can be rigorously justified thanks to the Mori–Zwanzig theory (10, 11). The latter also lays the foundation for the use of data-driven methods aimed at estimating the role of the unresolved scales of motion on the resolved ones (12). And, indeed, low-dimensional stochastic models have proved very valuable tools for understanding the statistical and dynamical properties of complex systems, e.g. in the area of Earth system science (13–18).

Either way, a well-known fact is that we currently observe a finite predictability horizon for systems of interest like, for example, the atmosphere (19, 20). This means that, when forecasting the future behavior of a dynamical system, the error that we make tends to increase the longer into the future we want to forecast (21).

To understand predictability and the methods commonly used to assess it, we look at the evolution of the system in the so-called phase space, that is the space of all the variables used to describe it. In the phase space usually we can identify a subset, namely a finite-size manifold of trajectories followed by the dynamical system over time and denoted by x(t), where x is the vector of observables that depends on time t. This finite-size manifold is also known as attractor [random attractor in the case of stochastic dynamical systems (22)].

The origin of this practical unpredictability may stem either from an intrinsic stochastic nature of the system or from the ignorance of the current state of the system and/or of the dynamic rule controlling its evolution (23), or from a combination of both. Furthermore, it is well known that even low-dimensional nonlinear systems may be difficult to forecast if they exhibit an exponential divergence of close-by states (19). In such cases, the unpredictability comes from our lack of precise knowledge of the current state of the system: If we knew exactly the initial conditions, we would always get the same future evolution. A common quantity used to characterize the predictability of a system is the maximum Lyapunov exponent $\lambda_{\text{max}}$, which measures the fastest possible rate of exponential divergence between two nearby trajectories in the phase space for an infinitely long time horizon. If we look at the evolution of two trajectories starting from two points close to each other, namely $x(t_{0})$ and $x(t_{0})+\delta x$, these will diverge exponentially fast as time progresses, until their distance will plateau around a value bounded by the diameter of the attractor (24). Even more instructive is to look at the evolution of a ball around the state x(t). From this, one can define a full Lyapunov spectrum, made of Lyapunov exponents equal in number to the variables used to describe the phase space. The Lyapunov exponents are mean logarithmic growth rates of the lengths of the principal axes of this ball evolving under the action of the dynamic rule (4, 5, 24, 25). The existence of at least one positive Lyapunov exponent is the hallmark of chaotic dynamical systems. Therefore, measuring predictability in dynamical systems can be reframed as computing the divergence of a system’s nearby trajectories over time.

It is important to highlight that the Lyapunov exponents are averaged quantities and the average is typically performed over a long trajectory. As a consequence, they serve as key indicators of the system’s global predictability (4, 25), where the term global refers to average properties of the system, hence to the average predictability of the system. While the average predictability of the system can be useful, theoretical curiosity as well as operational requirements and priorities indicate the need to investigate a local notion of predictability. Indeed, the stability properties of a dynamical system can be dramatically state-dependent (26). We then wish to understand the system’s predictability in a specific region of the phase space (we will refer to these local characteristics also as instantaneous, since they refer to a specific time) (27, 28). This notion has extreme relevance in the context of weather prediction (29) and key implications for devising efficient and accurate data assimilation methods (30).

To this end, local (or finite-time) Lyapunov exponents (28, 31–33) are useful quantities. However, their reliance on linearized dynamics prevents the characterization of the system outside the linear regime of perturbations. Acknowledging this limitation, finite-size Lyapunov exponents (34, 35) and nonlinear local Lyapunov exponents (36, 37) were subsequently proposed to quantify local predictability in the nonlinear regime, as they consider finite perturbations. Nonetheless, it is noteworthy that they both assume an exponential law for error growth. Although this is consistent with classic Lyapunov exponents within the linear regime, this assumption often does not hold in the nonlinear regime (i.e., for finite errors). These local indices have been applied successfully to various dynamical systems of varied complexity to gain insights into their predictability (32, 34, 36, 38), despite the aforementioned limitations.

Besides Lyapunov exponents and their variants, information theory also offers methods to quantify predictability (23, 39). Within this framework, various entropy-based indicators are usually employed as predictability measures for model-based prediction, with varying degrees of success (40–43). However, because these methods are model-based, they are unsuitable for observational datasets. Other indices from nonlinear time series analysis also use predictability measures such as sample entropy, permutation entropy, and others (44–46); nevertheless, they are only effective with time series or systems with relatively low dimensionality. To the best of the authors’ knowledge, no information theory method can infer local predictability information from high-dimensional observational data.

From the brief review of relevant literature just outlined, the above mentioned indices have several drawbacks. More specifically, Lyapunov exponents and their variants may not work well for nonlinear regimes, while information-theory-based methods may not be directly applicable to high-dimensional dynamical systems. In the next section, we leverage the recent framework proposed by Lucarini et al. (47), which uses recurrences to characterize local dynamical properties, to develop a local predictability index that differs from existing metrics, such as the Lyapunov exponents and their variants.

## A Local Data-Driven Approach

Lucarini et al., (47) introduced a novel framework to measure the local properties of dynamical systems. In particular, they combine extreme value theory (48) and the Poincaré recurrence theorem (2) to construct purely data-driven local dynamical indices, that can measure the local dimension of dynamical systems and their persistence at any point in time. Under the ergodic assumption, the Poincaré recurrence theorem guarantees that the system will visit an arbitrarily small neighborhood of any state again and again as time passes. In this framework, for one given state of interest $\zeta =x(t=t_{\zeta })$ in the phase space, its neighboring states, also known as recurrences, are used to characterize its local dynamical properties. This can be formalized by defining the negative logarithmic distance between ζ and all other states

[1] $$g\left(\zeta ,x(t)\right)=-\log\left[\text{dist}\left(x(t),\zeta \right)\right],$$

where the dist function can be any distance metric, and by subsequently defining a quantity called exceedance for the neighboring states (i.e., recurrences):

[2] u(ζ)=g(ζ,x(t))−s(q,ζ),∀g(ζ,x(t))>s(q,ζ).

In Eq. **2**, s(q,ζ) is a high threshold corresponding to the q-quantile of the series $g\left(\zeta ,x(t)\right)$, noting that having g(ζ,x(t)) above the threshold s(q,ζ) is a synonym of requiring a trajectory to fall within a neighborhood of ζ (defined as a ball).

Building on the above-defined quantities and leveraging extreme value theory, this framework introduces two local dynamical indices: The local dimension d and persistence Θ (see *Materials and Methods* for a detailed explanation and derivation), both of which are frequently associated with the predictability of dynamical systems (49–51). More specifically, high local dimension and low persistence are seen as an indication that the system is less predictable. The rationale for this is as follows. If a system requires more degrees of freedom (high d) to be described at a given point in the phase space, then its complexity is greater at that given point, making it possibly less predictable. Similarly, if a system leaves a neighborhood quickly (low Θ), then the dynamics in that neighborhood are fast and nonpersistent, an aspect that may indicate reduced predictability. While these arguments on predictability are qualitatively valid, and frequently correct, they approach predictability from a complexity (d), and mean residence time (Θ) perspective, without directly addressing predictability.

In this work, we address the predictability of dynamical systems by building upon the framework of local dynamical indices just outlined. In particular, we introduce a new quantity named “time-lagged recurrence” (also referred to as TLR) and denoted by $\alpha_{\eta }$. This new quantity uses neighboring states to monitor an ensemble of trajectories, and provides an indication in a statistical sense of the local predictability of dynamical systems. This is accomplished in a purely data-driven fashion, i.e. without requiring a model nor further simplifications on the data.

**Fig. 1.**
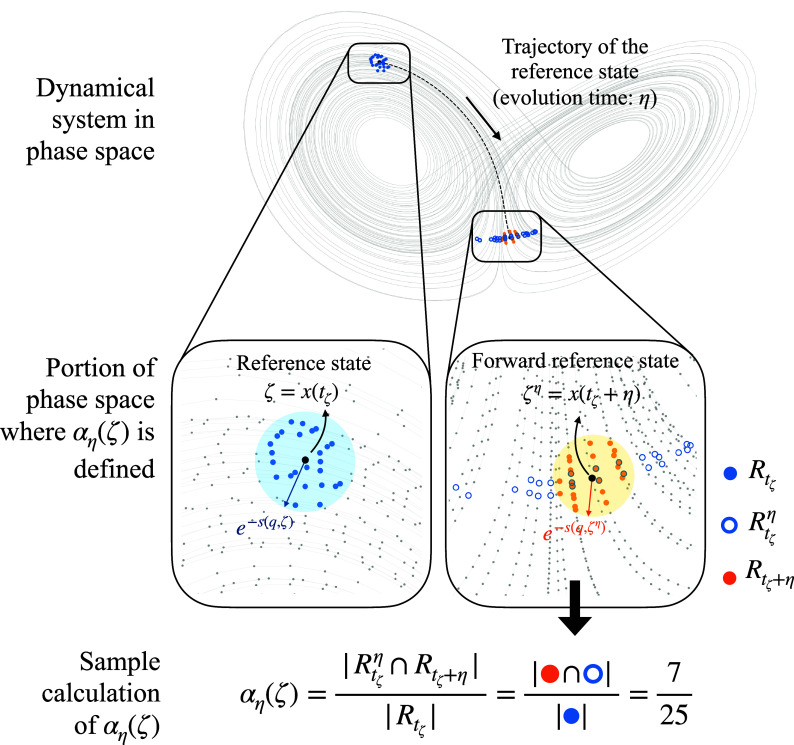
Schematic illustration of the computation of $\alpha_{\eta }\left(t\right)$, demonstrated in the phase space of the Lorenz-63 system. This figure presents all steps involved in computing the *time-lagged recurrence* for the reference state ζ at a forecasting horizon η, namely $\alpha_{\eta }\left(\zeta \right)$. The panels in the second row provide a zoomed-in view of the phase space region where $\alpha_{\eta }\left(\zeta \right)$ is defined. *Recurrences* ($R_{t_{\zeta }}$), *forward recurrences* ($R_{t_{\zeta }}^{\eta }$), and *forward-reference-state recurrences* ($R_{t_{\zeta }+\eta }$) are represented by solid blue dots, empty blue dots, and orange dots, respectively. The blue circle with radius $e^{-s(q,\zeta )}$ indicates the hypersphere used to define the neighborhood of the reference state, while the orange circle with radius $e^{-s(q,\zeta^{\eta })}$ corresponds to the forward-reference-state.

The computation of $\alpha_{\eta }$ also relies on the concept of recurrences and the quantities defined above in Eqs. **1** and **2**, with a schematic illustration presented in Fig. 1. More specifically, we identify the set of states—i.e., recurrences—$R_{t_{\zeta }}$ in the neighborhood of the reference state ζ, that is

[3] $$R_{t_{\zeta }}={\left\{x\left(t_{u_{i}}\right):u_{i}\left(\zeta \right)>0\wedge \left|t_{\zeta }-t_{u_{i}}\right|>w\right\}}_{i=1}^{N_{n}},$$

where $u_{i}\left(\zeta \right)$ represents the exceedances, defined in Eq. **2**, and with positive values indicating proximity to the reference state in phase space, $t_{\zeta }$ is the time that identifies the reference state ζ, $N_{n}$ is the number of recurrences (i.e., the number of temporally nonadjacent neighbors) at time $t_{\zeta }$, $t_{u_{i}}$ is the time corresponding to the exceedance $u_{i}$, and w is the Theiler window (52) that excludes temporal neighbors of ζ. We note that if w is chosen too small that cannot exclude temporal neighbors it will lead to spurious high values of $\alpha_{\eta }$.

After having identified the recurrences $R_{t_{\zeta }}$, to characterize the local predictability of ζ, we first need to estimate how neighboring states evolve after time η has passed from $t_{\zeta }$. To this end, we first evolve the states within $R_{t_{\zeta }}$ forward by time η (where η is also referred to as forecasting horizon), which leads to a set of new states, that we name forward recurrences

[4] $$R_{t_{\zeta }}^{\eta }=\left\{x\left(t+\eta \right)\;\;\;\forall x\left(t\right)\in R_{t_{\zeta }}\right\}.$$

These new states, that were evolved forward in time by η from time $t_{\zeta }$, are represented by the circles with blue contours in Fig. 1, and are denoted by using the superscript ${\left(\cdot \right)}^{\eta }$.

Once the forward recurrences have been defined (Eq. **4**), we need to define the forward reference state, that is, the reference state $\zeta =x(t_{\zeta })$ after time η, and denoted by $\zeta^{\eta }=x\left(t_{\zeta }+\eta \right)$. This forward reference state will have its own exceedances $u(\zeta^{\eta })$ made of $N_{n}$ elements $\{u_{i}\left(\zeta^{\eta }\right){\}}_{i=1}^{N_{n}}$, and we can compute the forward-reference-state recurrences

[5] $$R_{t_{\zeta }+\eta }={\left\{x\left(t_{u_{i}}\right):u_{i}\left(\zeta^{\eta }\right)>0\wedge \left|t_{\zeta }+\eta -t_{u_{i}}\right|>w\right\}}_{i=1}^{N_{n}},$$

in analogy to what was done for the reference state ζ in Eq. **3**.

With the three sets just introduced in Eqs. **3**–**5**, namely the recurrences $R_{t_{\zeta }}$ in Eq. **3**, the forward recurrences $R_{t_{\zeta }}^{\eta }$ in Eq. **4**, and the forward-reference-state recurrences $R_{t_{\zeta }+\eta }$ in Eq. **5**, we can define the local predictability index as follows:

[6] $$\alpha_{\eta }\left(\zeta \right)=\frac{|R_{t_{\zeta }}^{\eta }\cap R_{t_{\zeta }+\eta }|}{|R_{t_{\zeta }}|},$$

where |(·)| represents the number of elements within the set (·), and ∩ is the intersection of two sets. Since both $R_{t_{\zeta }}$ and $R_{t_{\zeta }+\eta }$ are defined using the same quantile-based thresholds, the number of elements in one set is equal to the number of elements in the other set, that is, $|R_{t_{\zeta }}\left|\;=\;\right|R_{t_{\zeta }+\eta }|\;=N_{n}$. In addition, given that $R_{t_{\zeta }}^{\eta }$ is defined from $R_{t_{\zeta }}$, the number of elements it contains is also equal to $N_{n}$. It follows that $0\leq \alpha_{\eta }\left(x\left(t\right)\right)\leq 1$. A value of $\alpha_{\eta }\left(\zeta \right)$ close to 1 indicates high probability for a neighbor state to remain close to the reference state. We interpret this as a high predictability, in a statistical sense, for state ζ at time $t_{\zeta }$, and forecasting horizon η, suggesting that most of its neighboring states stay close to the future trajectory of ζ as time evolves. Conversely, when $\alpha_{\eta }\left(\zeta \right)$ is close to zero, this implies that most of the initial neighbors have moved away from the trajectory of ζ after time η, indicating low predictability in a statistical sense.

The derivation of the new quantity $\alpha_{\eta }\left(\zeta \right)$ relies on the distance function dist and the quantile q, both of which are used to define recurrences. Although the Euclidean distance in phase space has been shown to be an appropriate choice for physical systems (47), the distance function can be adjusted depending on the prior knowledge of the system and the application context. The selection of the quantile q can be adapted for different application scenarios, and it reflects the local statistical predictability for different scales. This is not surprising as we are defining $\alpha_{\eta }$ using extreme value theory where quantile choice is always an important step. In *SI Appendix*, section 2, we show an example of this scale-dependent behavior using various q. This dependence on q can be seen as a favorable property as it allows for analyzing predictability at different scales.

In addition, the new quantity $\alpha_{\eta }\left(\zeta \right)$ is inherently connected to information theory, since estimating local predictability can be viewed as a process that quantifies the uncertainty or randomness within the system. In fact, if we take a conditional probability perspective, where P(A|B) represents the conditional probability of A given B, then we can reformulate the definition of $\alpha_{\eta }$ in Eq. **6** as follows:

[7] $$\alpha_{\eta }\left(\zeta \right)=P\left[x\left(t+\eta \right)\in R_{t_{\zeta }+\eta }|x\left(t\right)\in R_{t_{\zeta }}\right],$$

where $A=x\left(t+\eta \right)\in R_{t_{\zeta }+\eta }$ corresponds to recurrences of the reference state ζ that, after being propagated forward in time by η, belong to the forward-state neighborhood $\zeta^{\eta }$ (i.e., they are forward-reference-state recurrences), and $B=x\left(t\right)\in R_{t_{\zeta }}$ corresponds to the recurrences of the reference state ζ. We can further link $\alpha_{\eta }$ to information theory using the Shannon entropy, when defined—see *SI Appendix*, section 3.

The new predictability index has a number of advantages compared to existing predictability measures: i) it is purely data-driven, ii) it is local as it measures instantaneous properties, iii) it does not require simplifications to be made on the data, and iv) it is suitable for high dimensional datasets.

However, given that it uses future states, it cannot be readily used for real-time predictability analyses, for instance in the context of operational weather forecasting. We shall discuss this aspect in a later section and propose a practical way to address this limitation.

It is helpful here to emphasize some key differences to other data-driven ways of measuring local predictability properties. The classical algorithms proposed in ref. 53 to measure the Lyapunov exponents from available time series allow to estimate the growth rate of the n−dimensional volumes (for n smaller than or equal to the dimension of the phase space) defined by n+1 initially nearby points. The strategy there strongly relies on using delay coordinates in order to take advantage of Takens’ theorem (54) and reconstruct the key properties of the attractor. By selecting nearby points belonging to a region of interest, local properties can be extracted. Following such geometrical construction, one obtains that

[8] $$P\left[\;\text{dist}\left(x\left(t+\eta \right),\zeta^{\eta }\right)<\epsilon |\;\text{dist}\left(x\left(t\right),\zeta \right)<\epsilon \right]\approx e^{-\left(\sum_{\lambda_{j}>0}\lambda_{j}^{\zeta }\right)\eta },$$

where the exponent is proportional to the sum of positive local, finite-time, Lyapunov exponents. Clearly, ϵ is assumed to be small. After comparing Eqs. **7** and **8** it is tempting to deduce $\alpha_{\eta }\approx e^{-(\sum_{\lambda_{j}>0}\lambda_{j}^{\zeta })\eta }$ (see later discussion on specific numerical examples). Yet, a key difference between the two definitions exists when one applies them to actual data, because in Eq. **7** one considers neighborhoods of ζ and $\zeta^{\eta }$ having the same number of points (thanks to the use of quantiles in defining the threshold of the distance observables), so that such neighborhoods will in general have different radii (which are instead identical and equal to ϵ in Eq. **8**).

## Results and Discussion

We have computed the new metric $\alpha_{\eta }\left(\zeta \right)$ for systems of varying complexity to show its effectiveness. The following subsections provide a detailed discussion of the results for the Lorenz-63 attractor and climate data characterizing atmospheric circulation in the Euro-Atlantic sector. Additionally, a brief summary of $\alpha_{\eta }\left(\zeta \right)$ applied to other systems is presented in a subsequent section to illustrate its broad applicability. Following the discussion of results obtained from various systems, we propose a method for utilizing this index in real-time, addressing the issue introduced at the end of the previous section.

### Lorenz-63.

**Fig. 2.**
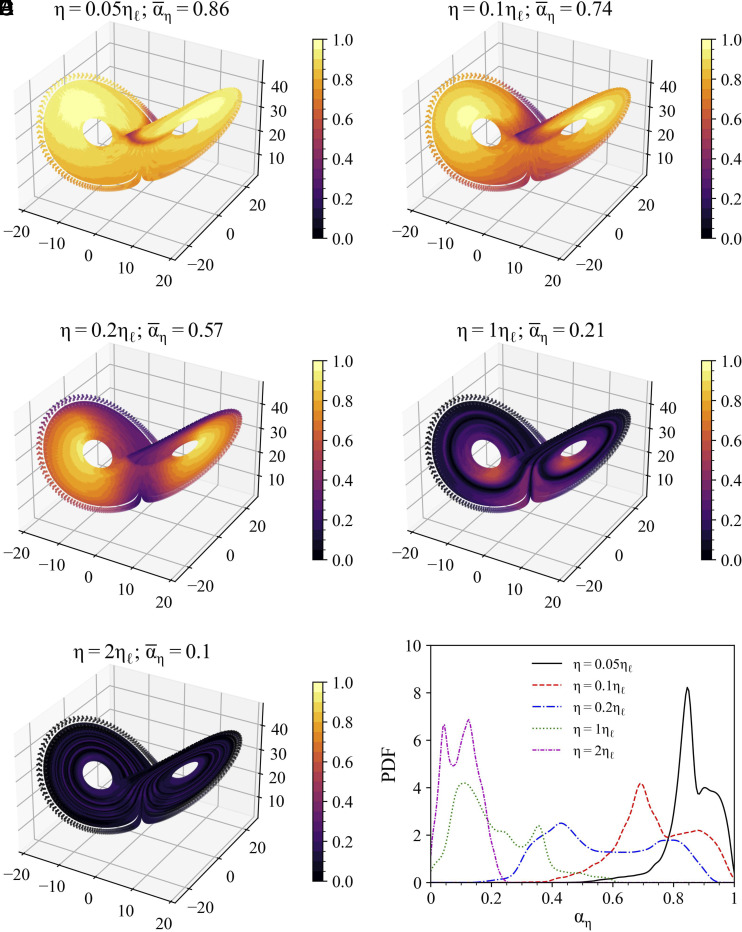
Distribution of $\alpha_{\eta }$ at different prediction horizons for the Lorenz-63 system. (*A*–*E*) Lorenz attractor colored by $\alpha_{\eta }$ values at five different forecasting horizons: $\eta =\left[0.05,0.1,0.2,1,2\right]\eta_{\ell }$, each corresponding to timesteps: L=[11,22,44,220,440]. (*F*) Probability distribution of $\alpha_{\eta }$ at the same five different forecasting horizons. The quantile q applied in this analysis is 0.99, and the Theiler window size w is set to 50 time steps.

We consider the Lorenz-63 attractor as an example of idealized dynamical system. The data for this test case is obtained from the numerical simulation of the Lorenz-63 system, with a time resolution of $5\times 10^{-}3$ over $1\times 10^{5}$ time steps, using the classical set of parameters that leads the system to be chaotic (see *SI Appendix*, section 1 for details). Fig. 2 shows the distribution of $\alpha_{\eta }$ for the Lorenz-63 system, for five different forecasting horizons: $\eta =\left[0.05,0.1,0.2,1,2\right]\eta_{\ell }$. The quantity $\eta_{\ell }$, that represents the Lyapunov time, is equal to 1.1 time units and is computed as the inverse of the maximum Lyapunov exponent of the Lorenz-63 system.

Fig. 2*A*–*E* depict the Lorenz attractor in the phase space colored by the values of $\alpha_{\eta }$, for the 5 different forecasting horizons considered. For each panel in Fig. 2*A*–*E*, the title on the top reports the forecasting horizon, and the average of the local predictability, namely ${\overset{¯}{\alpha }}_{\eta }$. We note how, as we increase the forecasting horizon, ${\overset{¯}{\alpha }}_{\eta }$ tends to decrease, as also depicted by the increasingly darker colors from Fig. 2*A*–*E*. This result is in agreement with what we shall expect—the longer the forecasting horizon, the less predictable the system. This conclusion is also summarized in Fig. 2*F*, that shows the distribution of $\alpha_{\eta }$ for the five different forecasting horizons: The two longest horizons display a distribution peaked on low values of predictability (∼0.2), while shorter horizons tend to display distributions peaked toward higher values of predictability. To further test the resilience of $\alpha_{\eta }$ to noise, we applied $\alpha_{\eta }$ to the Lorenz-63 system with stochastic diffusion terms for comparison (*SI Appendix*, Fig. S1). The results are similar to Fig. 2, while we observe small-scale fluctuations of $\alpha_{\eta }$ over the attractor and a more rapid drop in average predictability, as one shall expect.

These results confirm that the predictability of the Lorenz-63 system is closely related to the local reference state in the phase space and the forecasting horizon. In addition, the phase-space distribution of $\alpha_{\eta }$ shows some interesting features, namely: for short forecasting horizons the region where the Lorenz-63 attractor switches lobes seems to have low predictability (Fig. 2*A*), while for longer forecasting horizons, also the wings of the attractor become less predictable (Fig. 2 *B* and *C*). These findings are consistent with previous studies on local predictability of Lorenz systems (21, 27). Remarkably, for forecasting horizons twice the Lyapunov time, the overall system loses predictability (Fig. 2*E*).

**Fig. 3.**
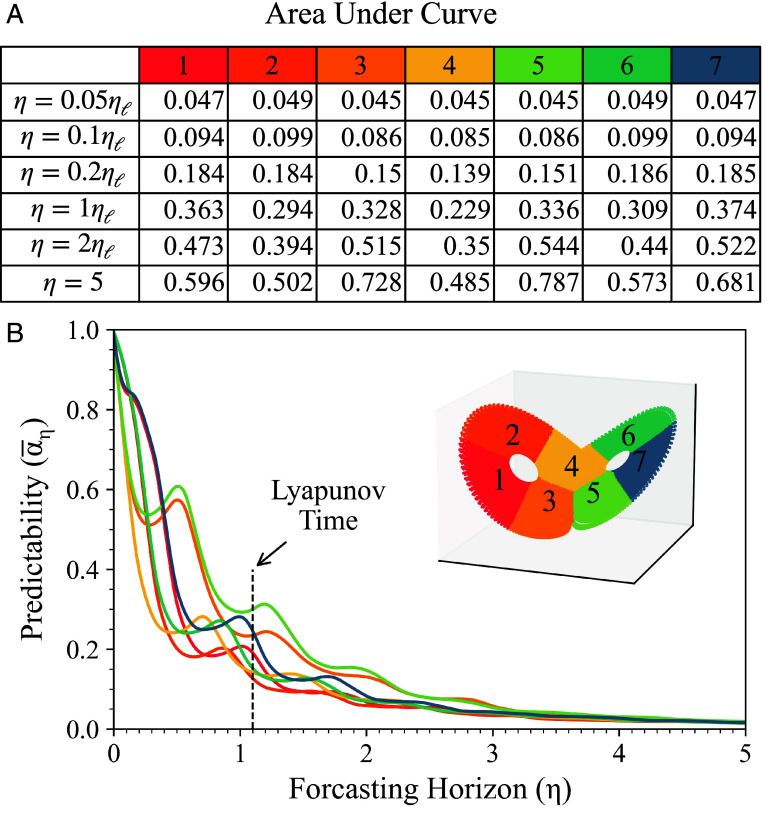
Temporal and phase-spatial variations of $\alpha_{\eta }$. (*A*) Area under the curve for different forecasting horizons and phase space regions, calculated from the time series shown in panel (*B*). Each cell color corresponds to the respective cluster in (*B*). The evolution of $\alpha_{\eta }$ averaged across seven clusters of states on the Lorenz-63 attractor. *Inset*: Lorenz-63 attractor colored to represent seven clusters obtained from K-means algorithm, each labeled with corresponding numbers.

To further show how $\alpha_{\eta }$ reveals phase-space and time features that can be used for understanding the predictability of dynamical systems, we present the evolution of the average $\alpha_{\eta }$ for seven different clusters (or regions) of the Lorenz-63 attractor. These clusters are depicted in Fig. 3, and are obtained by applying a K-means clustering method to the Lorenz-63 data used for Fig. 2, similar to the one adopted in ref. 55. Interestingly, the clustering identifies portions of the attractor in the phase space that seem to match some of the observations made for Fig. 2. In particular, cluster 4 represents the region where trajectories are switching lobes, clusters 1, 2, 6, and 7 represent the wings of the attractor, while clusters 3 and 5 represent intermediate regions that contain features from the five clusters just described. We note how, as expected, cluster 4 has the lowest short-term predictability among the regions considered, with a sudden drop at short forecasting horizons (yellow line). The wings of the attractor follow, with clusters 2 (dark orange) and 6 (green) being the least predictable after cluster 4, immediately followed by clusters 1 (red) and 7 (blue). Clusters 3 and 5, after a sudden predictability drop similar to the one experienced for cluster 4, maintain relatively good predictability for larger forecasting horizons compared to all other clusters. This is confirmed, if we look at the area under each curve (AUC) reported in Fig. 3, that represent the average predictability of each cluster with respect to the forecasting horizons, with clusters 3 and 5 having the highest average predictability, and cluster 4 the lowest.

We also note that, despite the general trend of predictability decreasing as the prediction horizon increases, $\alpha_{\eta }$ does not always decrease monotonically. There are instances showing returns of predictability, as also reported in ref. 56, and as reflected more clearly in Fig. 3*B*; see also discussion above regarding the relationship between the definitions provided in Eqs. **7** and **8**.

We additionally compare our results with the nonlinear local Lyapunov exponent (NLLE) (36, 37), an established index used to quantify local predictability that we find closely relevant, as reported in *SI Appendix*, section 4. The results for the NLLE display some notable differences with our local predictability index while also showing some similarities (*SI Appendix*, Fig. S6). These differences are arguably due to the use of different perspectives in defining predictability. The NLLE framework focuses on the exponential error growth rate, while our method is rooted in the concept of recurrences in dynamical system theory. We acknowledge that both methods have their own caveats: NLLE predefines the rule of error growth, while our method neglects the information on trajectories that have left the neighborhood. To overcome this, we propose an extension of our predictability index by accounting for the information given by the distance of all the neighboring trajectories—i.e., both those that remained close to the reference state and those that departed from it, as shown in *SI Appendix*, section 5.

**Fig. 4.**
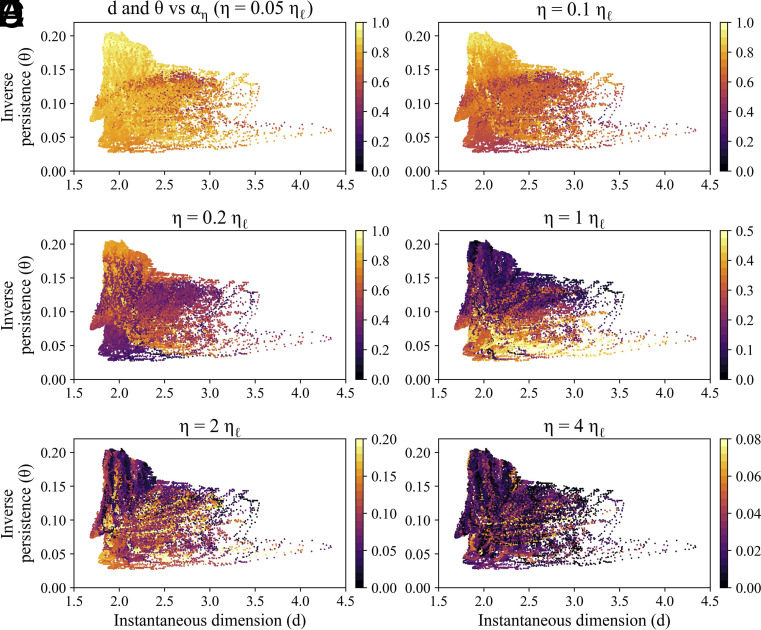
Comparative analysis of $\alpha_{\eta }$ with local dynamical indices for the Lorenz-63 system. (*A*–*F*) Scatter plots of local dimension versus local inverse persistence, where each dot represents one state. All states are colored based on $\alpha_{\eta }$ values for $\eta =\left[0.05,0.1,0.2,1,2,4\right]\eta_{\ell }$. Note that the color scale is different in the last three panels in order to improve readability.

Furthermore, we evaluated the relationship of the previously introduced local dynamical indices, d and Θ, to predictability, by comparing them against $\alpha_{\eta }$. In Fig. 4, we show scatter plots of the local predictability index $\alpha_{\eta }$ versus the local dimension d and the inverse persistence θ. We note that the intuition that high dimension and low persistence means low predictability does not hold for the case of the Lorenz-63 system. Specifically, states on the two wings of the Lorenz attractor exhibit low persistence (high values of θ), indicating that these states are likely to leave the neighborhood immediately (50). However, they also demonstrate high predictability over short forecasting horizons. This is not surprising when considering the dynamics on the wings of the Lorenz attractor—although these states may leave the neighborhood quickly, their neighboring trajectories do not diverge significantly, making them predictable in short forecasting horizons.

We show, in the next section, that, instead, for the real-world data considered, the agreement between the interpretation of the d-θ space and $\alpha_{\eta }$ seems to hold.

### Atmospheric Circulation.

In addition to the test case presented for idealized systems, we computed $\alpha_{\eta }$ using real-world data. In particular, we focused on studying the predictability of large-scale atmospheric circulation patterns in the Euro-Atlantic sector (see *Materials and Methods* for details). In this context, we consider the Z500 map for each day as a state along the system’s trajectory in the approximated phase space. We emphasize that this region serves as an ideal testbed to validate our predictability index due to its extensive body of relevant research, especially considering that no state-dependent predictability index is directly comparable for high-dimensional datasets.

In the Euro-Atlantic sector, the definition of weather regimes offers a direct way of classifying atmospheric states. Weather regimes are defined as recurrent and quasi-stationary large-scale circulation patterns (57, 58). Despite being a coarse characterization of atmospheric variability, weather regimes are found to be associated with conditions that are specifically favorable for certain types of weather extremes (59), and they have exhibited varying levels of predictability in real-time forecasting (60). In this work, we have adopted the four-regime definition widely used for scientific research (61), although various definitions for weather regimes exist (61, 62). Based on this definition, all extended wintertime (DJFM) Z500 daily patterns are classified into five different categories including NAO+, Atlantic Ridge (AR), NAO−, Scandinavian Blocking (SB), and no regime, using a method slightly adapted from ref. 63 (see *SI Appendix*, section 6 for details).

**Fig. 5.**
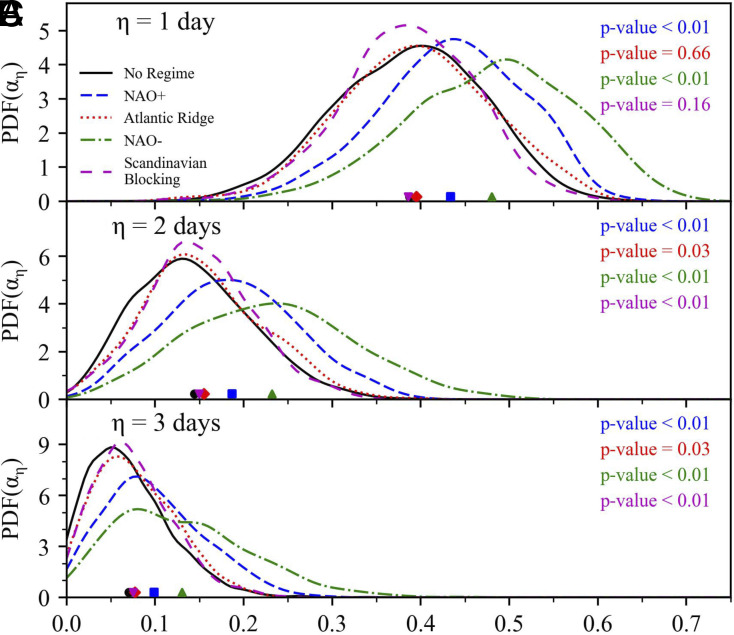
Predictability analysis of North Atlantic wintertime weather regimes. (*A*–*C*) Probability distribution of $\alpha_{\eta }$ for four weather regime and no regime: NAO+ (blue dashed line), Atlantic Ridge (red dotted line), NAO− (green dot-dashed line), Scandinavian Blocking (magenta loosely dashed line), and no regime (black line), for η=1 to 3 d. The markers on the x-axis show the mean value of $\alpha_{\eta }$ for different categories. The statistical significance is evaluated between the distribution of each weather regime and that of no regime using the Kolmogorov–Smirnov test. The quantile q applied in this analysis is 0.99, and the Theiler window size w is set to 7 d.

Fig. 5 shows the distribution of $\alpha_{\eta }$ across all five categories for η=1 to 3 d. The results indicate that the NAO− regime has the highest predictability, followed by NAO+, with the remaining two regimes showing no significant difference from having no regime for η=1 d. For η=2 and 3 d, NAO+ and NAO− still maintain notably higher mean values compared to other categories, with the no regime category having the lowest values, confirming that large scale weather regimes are a primary source of predictability in the Euro-Atlantic region. These results are in excellent agreement with previous investigation dedicated to the Euro-Atlantic sector: NAO− exhibits stronger predictability than other regimes, while the blocked zonal regimes (SB and AR) are notoriously unpredictable (50, 60, 64). Additionally, we find further confirmation of the general fact that blockings are associated with anomalously high instability compared to zonal flow conditions (65, 66). These findings further validate the effectiveness of our index, supported by both the underlying physical explanations and the model-based prediction results. We note that the analysis just presented can be extended to any other variables and domains, with a key region of interest being the tropical Indo-Pacific, given the complex interaction of different modes of variability (67).

**Fig. 6.**
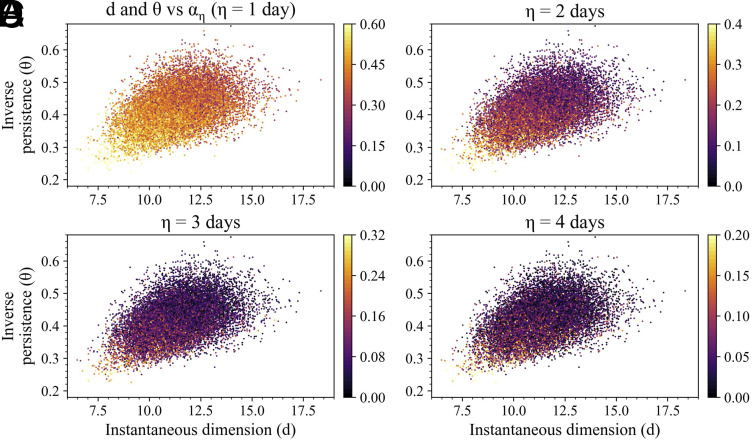
Comparative analysis of $\alpha_{\eta }$ with local dynamical indices for Z500 patterns in the Euro-Atlantic sector. (*A*–*D*) Scatter plots of local dimension versus local inverse persistence, where each dot represents one daily Z500 pattern. All states are colored based on $\alpha_{\eta }$ values for η=1 to 4 d. Note that the color scale is different in each panel in order to improve readability.

Fig. 6 illustrates scatter plots of local dimension d vs. inverse persistence θ for all the atmospheric states, colored by the predictability index $\alpha_{\eta }$, for different prediction horizons (η) ranging from 1 to 4 d. The overall predictability drops as η increases, as expected due to the highly chaotic nature of atmospheric circulation. Notably, states in the lower-left corner of the d-θ scatter plot space consistently exhibit higher-than-average predictability, regardless of the value of η. This aligns with previous results suggesting that states with low local dimension and high persistence are more predictable. From a dynamical system theory perspective, a lower local dimension indicates a simpler dynamics, while higher persistence means that a state is likely to remain similar to its current configuration for a longer time. This result demonstrates the effectiveness of the proposed predictability index for high-dimensional systems.

### Other Systems.

**Fig. 7.**
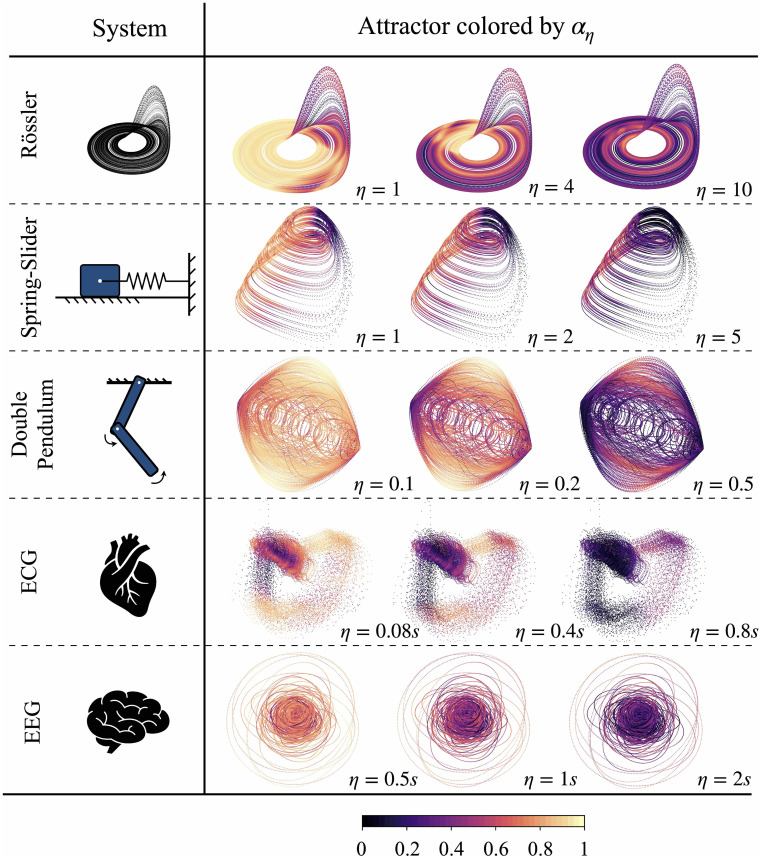
Summary of $\alpha_{\eta }$ applied to various systems, including another canonical dynamical system (the Rössler attractor), simulated slow earthquakes (spring-slider model), a classical physics problem (the double pendulum), and real-world biological data (observational ECG and EEG data). The forecasting horizon η is annotated at the *Bottom-Right* corner of each attractor. All the attractors presented here are three dimensional for demonstration purposes, but their corresponding $\alpha_{\tau }$ are computed in higher-dimensional spaces, as detailed in *SI Appendix*, section 7. The quantile q applied in all analysis is 0.99, and the Theiler window size w is set to 50 time steps.

In addition to the Lorenz-63 system and climate data, in Fig. 7, we demonstrate the applicability of $\alpha_{\eta }$ across a diverse range of systems. These include another canonical dynamical system (Rössler attractor), simulation data for slow earthquakes (spring-slider system), a classical physics problem (double pendulum), and real-world biological data (observational ECG and EEG data). The stochastic spring-slider system was originally introduced to simulate slow earthquakes, which feature a random attractor governed by stochastic differential equations, as detailed in ref. 16. The final three examples use the same dataset as that of ref. 68, with the double pendulum data generated through numerical simulation, while the other two are obtained from observational data.

The results for the Rössler attractor show a certain degree of similarity to those of the Lorenz-63 system: The Lorenz-63 system experiences a significant drop in predictability before lobe transitions, whereas the Rössler attractor exhibits a similar phenomenon prior to bursting events in the z-direction. We also note that the region following the bursting events exhibits markedly higher predictability, as the trajectories passing through this region will oscillate predictably in the x-y plane until reentering the region prior to the bursting events. In the case of the spring-slider system, there is high predictability during the almost linear interseismic period. The time-lagged recurrence index starts decreasing when the slip rate of the slider increases. The fact that the onset of the rupture is marked by high unpredictability is in line with the idea that it is a difficult task to predict the final size of the event at the onset of the slipping phase. For the attractor of the double pendulum, we note that two endpoints of the attractor show noticeably lower predictability, whereas the transitional regions between them show relatively higher predictability, particularly for the outer parts of the attractor. In the two real-world biological data examples, we also observe that $\alpha_{\eta }$ reveals variability in predictability within the phase space across different forecasting horizons. These insights can improve the analysis of such systems and deepen our understanding of their underlying dynamics. The applications to these diverse systems collectively demonstrate that this method has broad applicability and effectiveness.

### A Note on Real-Time Predictability.

Our findings demonstrate the agreement of $\alpha_{\eta }$ with known results, positioning it as a viable index for analyzing the local predictability of dynamical systems. Yet, one key point remains outstanding: $\alpha_{\eta }$, as is, cannot be used for real-time applications, as it requires the future state of the system. However, since $\alpha_{\eta }$ quantifies local predictability information in the phase space, for a given unprecedented state $\zeta^{\prime }$, we can compute the average $\alpha_{\eta }$ of its analogues (i.e., similar states to $\zeta^{\prime }$ that appeared in the past). The number of analogues can be set once again using a quantile threshold, but this could be different from the one used to define the local properties. In practice, if we consider $N_{a}$ analogues, indicated by $\{x\left(t_{a_{i}}\right){\}}_{i=1}^{N_{a}}$, with $t_{a_{i}}$ being the time at which the analogue number $a_{i}$ occurred, the predictability index proxy is

[9] $${\hat{\alpha }}_{\eta }\left(\zeta^{\prime }\right)=\frac{1}{N_{a}}\sum_{i=1}^{N_{a}}\alpha_{\eta }\left(x\left(t_{a_{i}}\right)\right)$$

This can be used for real-time predictability analyses of dynamical systems. For instance, in the context of weather and climate applications, we can identify the analogues of today’s data, and compute the predictability index proxy ${\hat{\alpha }}_{\eta }\left(\zeta^{\prime }\right)$ using Eq. **9**. This will give us the real-time predictability of the system as of today, thereby allowing us to adopt the index for real-time applications. An example of real-time applications is presented in *SI Appendix*, section 8, where we show an application for the Lorenz-63 system, with the real-time predictability proxy ${\hat{\alpha }}_{\eta }\left(\zeta^{\prime }\right)$ successfully capturing the phase-space and time features of the predictability.

## Conclusions

This work complements the rich body of work on the pivotal topic of predictability of dynamical systems, from the pioneering works of Poincaré and Lorenz, to modern dynamical system theory and its applications to real-life complex systems, especially in the context of weather and climate prediction. In particular, we provide a time-lagged recurrence index that can infer state-dependent predictability from high-dimensional observational datasets, overcoming the curse of dimensionality that affects more traditional approaches. While our approach has resemblances with classical data-driven methods used for performing Lyapunov analysis on general systems (53, 69), the local predictability index differs from these methods and is able to describe flexibly key dynamical features of complex systems. We applied this index to dynamical systems of varying complexity, ranging from idealized low-dimensional systems to real-world high-dimensional atmospheric reanalysis datasets and we showcased its effectiveness. In particular, our findings confirm that when in the Euro-Atlantic sector of the mid-latitudes a blocking is present, the predictability of the atmosphere is typically anomalously low. We further showed how the new index reveals the scale-dependent nature of predictability by using different quantiles, and how it can be used in real-time problems. Our approach also addresses the ambiguity in terms of predictability that surrounds the two local dynamical indices, namely dimension d and persistence Θ, providing a framework that directly addresses predictability.

We conclude this work with a note. The definition of $\alpha_{\eta }$ does not require η>0. Therefore, $\alpha_{\eta }$ can be computed backward in time using η<0. In this case, $\alpha_{\eta }$ characterizes the dynamics of the system as it approaches the reference state of interest, and it provides information that is entirely different from the forward-in-time analysis presented so far, using η>0. Indeed, the backward predictability analysis can be used for understanding the dynamical pathways leading to extreme events as well as other phenomena in dynamical systems (70, 71). As a methodological study, we consider such in-depth analysis to be beyond the scope of this work, but we believe it may shed new light on various scientific domains.

## Materials and Methods

### Local Dynamical Indices.

In the framework proposed by Lucarini et al. (47), two dynamical indices, the local dimension d and persistence Θ, were introduced to characterize the local properties of dynamical systems. To compute these indices, we first consider the negative logarithmic returns $g\left(\zeta ,x(t)\right)$, as defined in Eq. **1**, which quantifies proximity to a given state ζ. The exceedance u(ζ), introduced in Eq. **2**, is defined for the neighboring states (i.e., recurrences) of ζ and measures how much $g\left(\zeta ,x(t)\right)$ exceeds a high quantile q. According to extreme value theory (72, 73), assuming the independence of the exceedances, the cumulative probability distribution F(u,ζ) follows the exponential member of the generalized Pareto distribution (GPD), that is

[10] $$F\left(u,\zeta \right)∼\exp\left[-\frac{u(\zeta )}{\sigma (\zeta )}\right].$$

The parameter σ(ζ), which is the scale parameter of the distribution, depends on the state ζ in phase space and can be used to derive the local dimension via d(ζ)=1/σ(ζ) (47). In practice, the local dimension is computed by explicitly fitting an exponential distribution to the exceedances u(ζ); see discussion in refs. 74 and 75. The estimate of the attractor’s dimension derived from d(ζ) seems to be more efficient than previous methods based on evaluating correlation integrals (76, 77). The other local index, persistence (Θ), is defined as the inverse of the extremal index 0≤θ≤1 (78, 79), a dimensionless parameter that measures the inverse of the length of clustering of extremes. In this context, extremes are defined as the *recurrences* within the neighborhood around a given state in phase space. Therefore, persistence (Θ) estimates the average number of consecutive recurrences, namely the time steps for the system to resemble a given state in its neighboring phase space.

This framework is the basis for the predictability index proposed in this work, and introduced in the main text above.

### 500 hPa Geopotential Data in the Euro-Atlantic Sector.

In this study, the atmospheric circulation dynamics in the Euro-Atlantic sector (80^°^W to 50^°^E, 22.5^°^N to 70^°^N) served as a testbed to evaluate the applicability of $\alpha_{\eta }$ on a real-world high-dimensional dataset. We used the state-of-the-art ERA5 reanalysis data (80) from 1979 to 2022, with a spatial resolution of 0.25^°^. The daily mean 500 hPa geopotential height (Z500) was selected as the representative variable, which has been frequently used to characterize atmospheric circulation dynamics in the mid-latitudes (58, 81–84). For this spatiotemporal system, we treat each daily spatial map of Z500 as an individual state, and the corresponding space can be regarded as an approximation to the phase space. This approach is widely adopted in practical applications involving real-world complex systems because accessing all variables of a system to construct its full phase space is often impossible. Instead, we can focus on a subset of available observables to approximate the system’s dynamics. In this way, the evolution of atmospheric circulation patterns can be viewed as a trajectory within this high-dimensional space, thereby enabling the application of $\alpha_{\eta }$ to assess its state-dependent predictability.

## Supplementary Material

Appendix 01 (PDF)

## Data Availability

The code for computing the local predictability index and the data used in this study are available at https://github.com/MathEXLab/TLR (85). The ERA5 reanalysis data used in this study are available at https://cds.climate.copernicus.eu/#!/search?text=ERA5&type=dataset (80) The data used for the double pendulum, ECG, and EEG are from ref. 68.
