# Supplementary material for: Time-lagged recurrence: A data-driven method to estimate the predictability of dynamical systems
Source: Proc Natl Acad Sci U S A. 2025 May 16;122(20):e2420252122. doi: 10.1073/pnas.2420252122 (PMC12107190; doi:10.1073/pnas.2420252122)
Supplement: Supplementary file 1 — Appendix 01 (PDF) [file pnas.2420252122.sapp.pdf]

Supporting Information for

Time-Lagged Recurrence: a data-driven method to estimate the predictability of dynamical systems

Chenyu Dong, Davide Faranda, Adriano Gualandi, Valerio Lucarini, and Gianmarco Mengaldo

Gianmarco Mengaldo.
E-mail: mpegim@nus.edu.sg

This PDF file includes:

Supporting text
Figs. S1 to S10
Tables S1 to S2
SI References

Supporting Information Text

1. Lorenz system and its stochastic version

The Lorenz-63 system (1) was originally derived from a simplified model of the atmospheric convection process and has been widely used as an example to study chaotic dynamical systems. Its governing equations can be written as:

$$\frac{dx}{dt} = \sigma(y-x), \quad [1]$$

$$\frac{dy}{dt} = x(\rho-z)-y, \quad [2]$$

$$\frac{dz}{dt} = xy-\beta z. \quad [3]$$

In this study, we adopt the classical parameters of the Lorenz system that exhibit deterministic chaos, where $(\sigma, \rho, \beta) = (10, 28, 8/3)$. Observational data are obtained through numerical simulation with a temporal resolution of $dt = 0.005$ over 10^5 time steps.

Additionally, we test the impact of noise on our predictability index by considering the Lorenz-63 system with diffusion terms. The corresponding equations can be written as:

$$\frac{dx}{dt} = \sigma(y-x)+\eta dW_t^1, \quad [4]$$

$$\frac{dy}{dt} = x(\rho-z)-y+\eta dW_t^2, \quad [5]$$

$$\frac{dz}{dt} = xy-\beta z+\eta dW_t^3. \quad [6]$$

where dW_t^j, $j = 1, 2, 3$ indicate increments of three independent Wiener processes, and η modulates the intensity of the stochastic perturbations. In Supplementary Fig. S1, we show the predictability index for the Lorenz system with stochastic perturbations, using the same numerical simulation settings as in Fig. 2, with the value of $\eta = 1$. We note that the Supplementary Fig. S1 is similar to Figure 2 for the five different forecasting horizons considered. However, we observe small-scale fluctuations of α_η, which corresponds to the characteristics of the stochasticity within the system. In addition, the value of α_η drops more rapidly on average as expected, since the stochastic nature will make the system less predictable in general.

2. The scale-dependent nature of predictability

Just like other local indices, the computation of α_η requires a quantile parameter q that defines *recurrences*, indicating the number of *recurrences* for each state used in the analysis. For local dimension d and inverse persistence θ, they are found to be stable within a certain range where the threshold is neither too high, making *recurrences* insufficient to fit the generalized Pareto distribution, nor too low, leading to other distributions (2). For α_η, although we do not need to fit the generalized Pareto distribution, we are still interested in how the value of q will affect the results.

Different values of q will result in different sizes of the neighborhood. Therefore, We interpret the predictability index derived from different q actually representing local predictability at different scales. The scale-dependent nature of predictability has been widely recognized in previous studies (3–5). For instance, in the linear regime, the maximum Lyapunov exponent denotes predictability for dynamical systems. However, when the error is finite (outside the linear regime), this is no longer sufficient because nonlinear dynamics play a more important role in predictability.

In this paper, all the results displayed in the main text use $q = 0.99$, which means the closest one percent of states to the reference state are selected as *recurrences*. Figure S2 and S3 show the results for $q = 0.98$ and $q = 0.995$, respectively, following exactly the same settings as Figure 2. We note that using different q values yields similar overall results, with the most prominent features being noticeable in each case. However, we also observe some differences: for larger q values ($q = 0.995$) , small-scale stripes on the attractor are visible, whereas for relatively smaller q values, no such patterns exist and the variation of α_η in phase space appears smoother. This confirms the close relationship between predictability and scale, indicating that the choice of q should be made according to specific application needs.

In Figure S4, we further tested a wider range of q values for their influence on the value of α_η. Four states at different locations on the Lorenz-63 attractor are shown in Fig. S4 (a), representing the wing, near the fixed point, before lobe transition (before the intersection of the two wings), and after lobe transition (after the intersection of the two wings). The α_η computed from q ranging from 0.98 (2000 *recurrences* for dataset with length of 10^5) to 0.9999 (10 *recurrences*) are demonstrated in Fig. S4 (b-e). We can see that although the overall differences given by different q values are not significant, there are still noteworthy distinctions that confirm the scale-dependent nature of predictability.

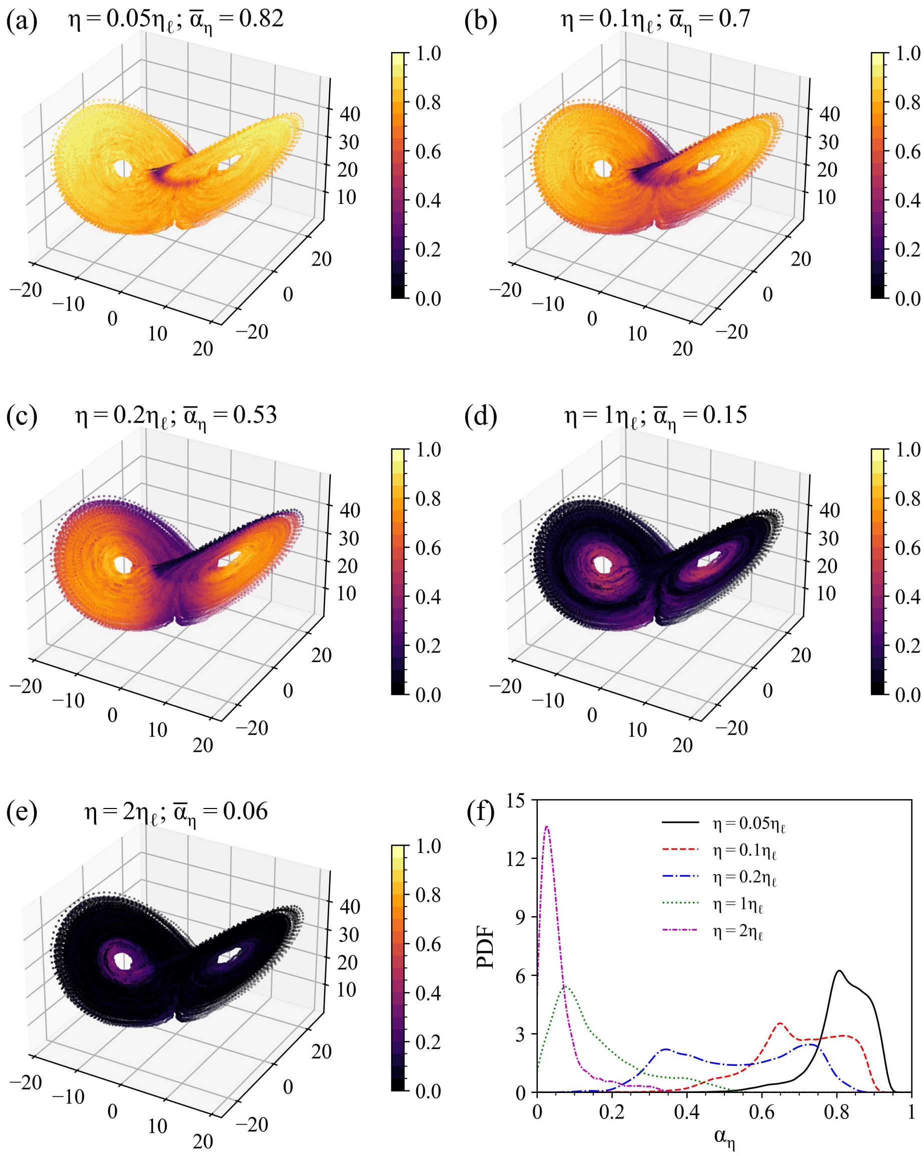

Fig. S1. Distribution of α_η at different prediction horizons for the stochastic Lorenz-63 system. Same as Figure 2, but for the stochastic version of the Lorenz-63 system. The quantile q applied in this analysis is 0.99, and the Theiler window size w is set to 50 time steps.

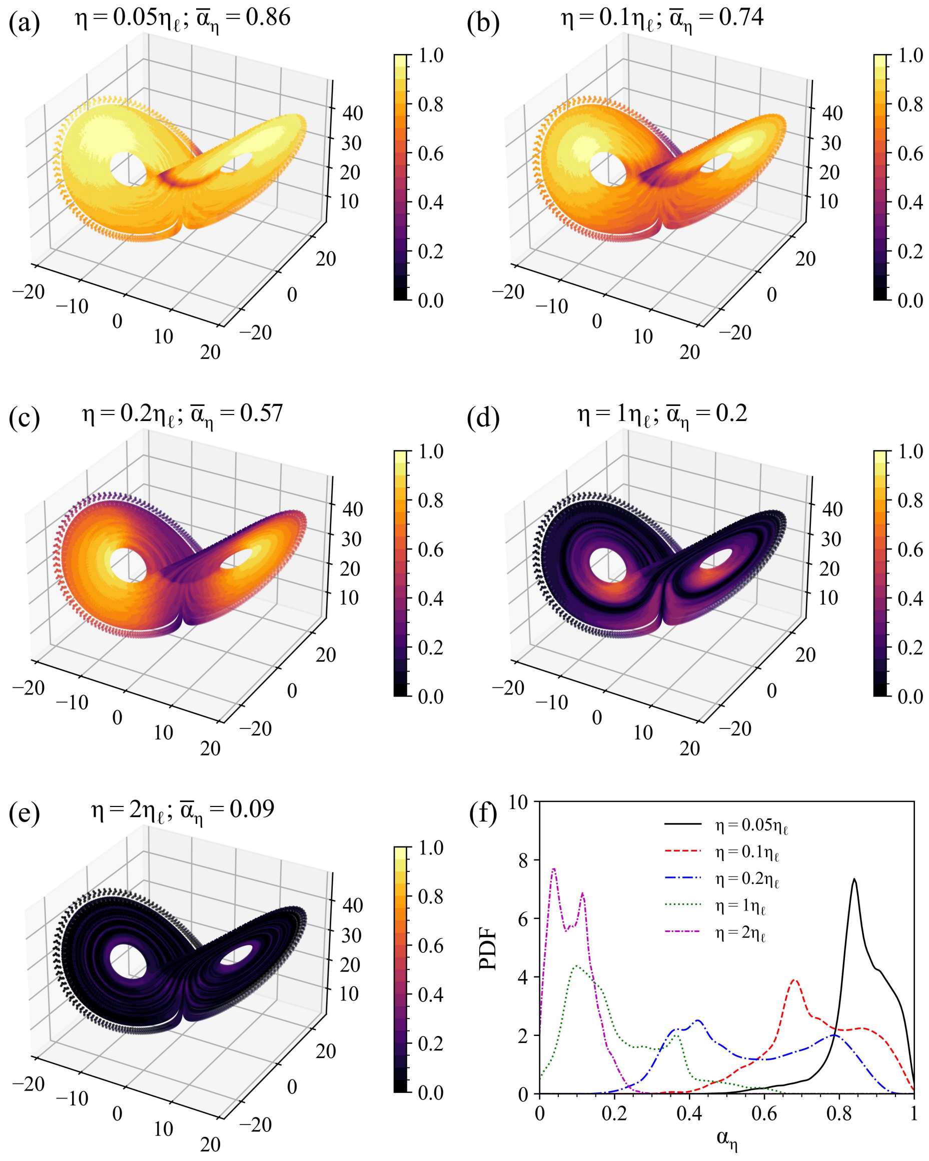

Fig. S2. Comparative analysis of the impact of different quantile choices (q) on α_t. Same as Fig. 2, but for $q = 0.98$.

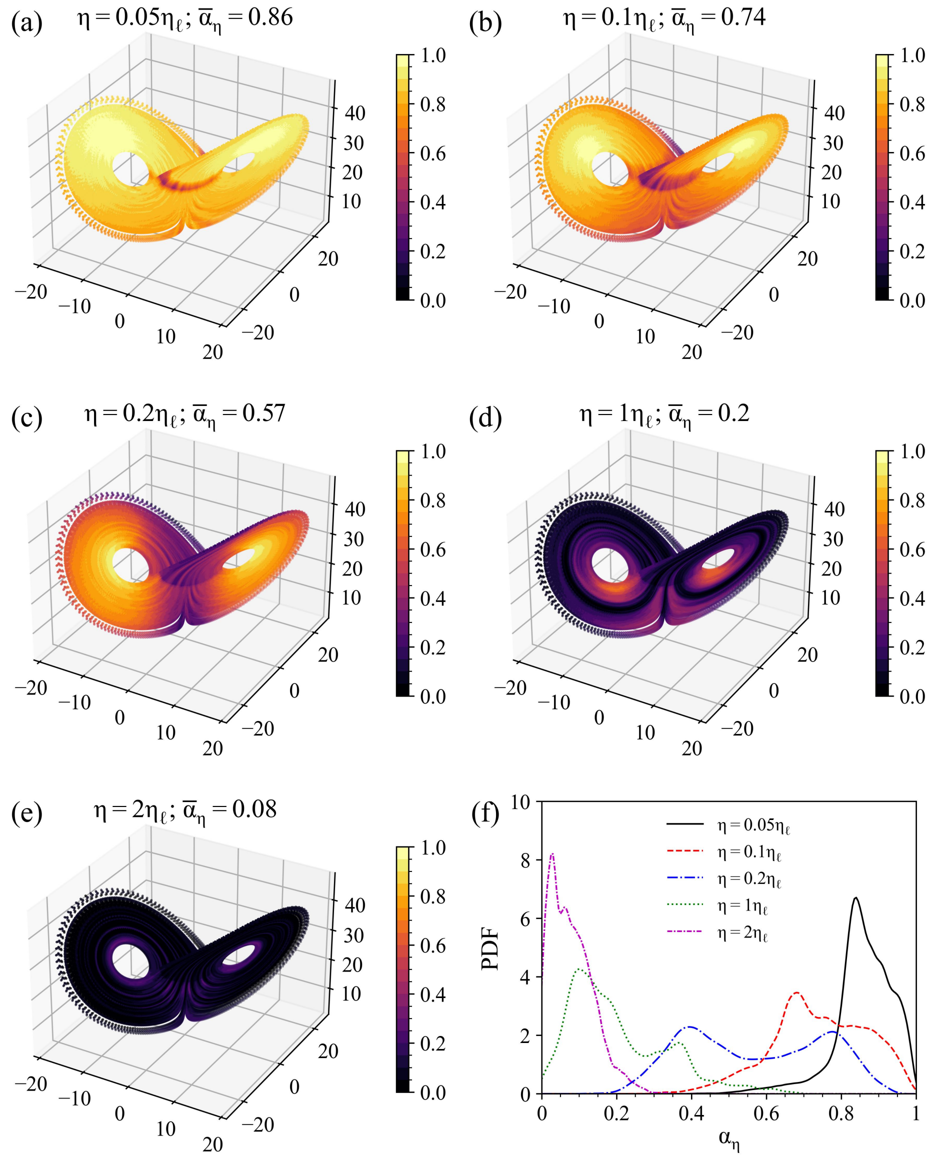

Fig. S3. Comparative analysis of the impact of different quantile choices (q) on α_t. Same as Fig. 2, but for $q = 0.995$.

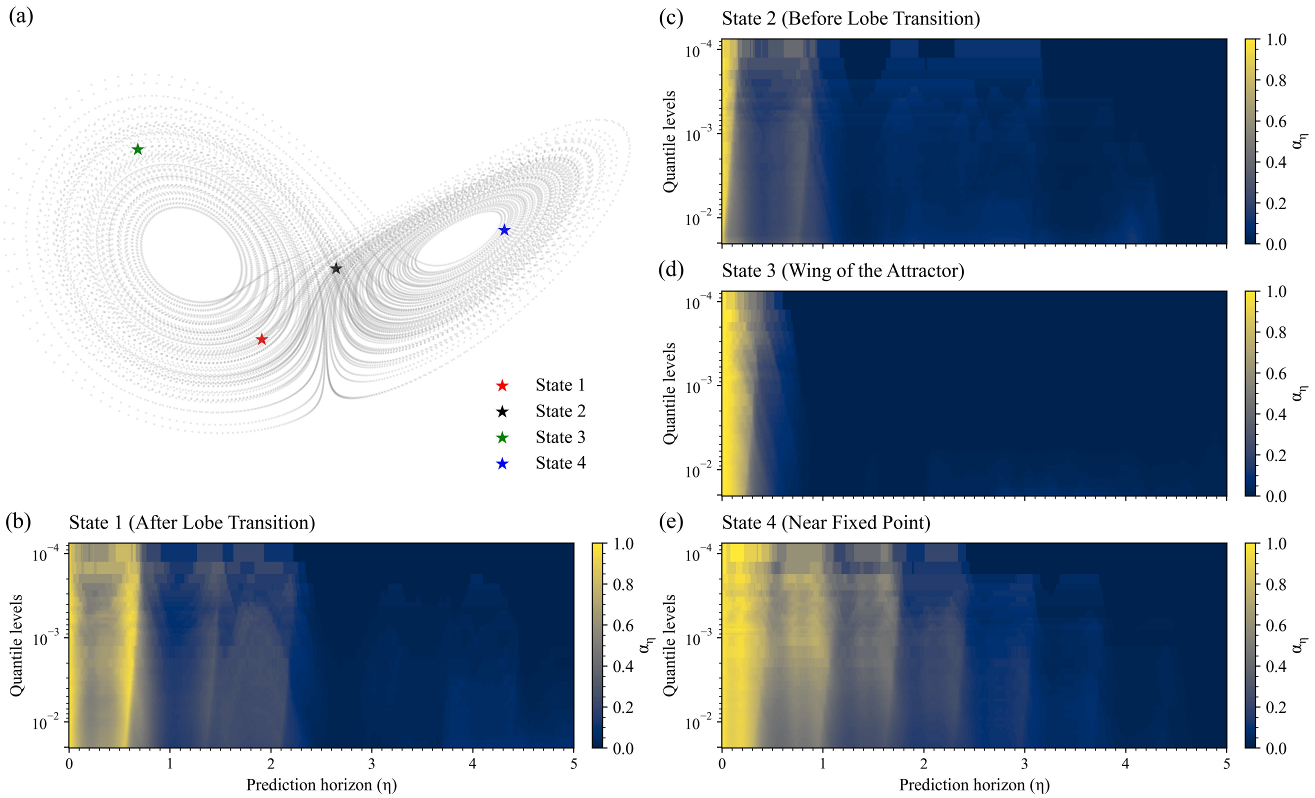

Fig. S4. Predictability analysis for different q values. (a) Illustration of the locations of the states considered in the Lorenz-63 phase space. (b-e) Heatmaps of α_η for four states located at the wing, near the fixed point, before lobe transition (before the intersection of the two wings), and after lobe transition (after the intersection of the two wings), respectively.

3. Information theory-based extension of α_η

In this section, we propose a link between the Shannon entropy, and our predictability index α_η. Local predictability of a dynamical system can be interpreted as the divergence rate of neighboring trajectories. As shown in step 1 of Fig. S5, we can adopt the same definitions of R_{t_ζ} and $R^\eta_{t_\zeta}$ as in α_η, and use second-order neighbors to characterize the divergence of states within $R^\eta_{t_\zeta}$ (step 2, in Fig. S5). Here, second-order neighbors are defined as the recurrences of all states within R_{t_ζ} and $R^\eta_{t_\zeta}$.

Let us set R_{t_ζ}, and recall that it contains N_n elements (states). Now, let us assume that for each of these elements, N_m second-order neighbors are selected, where N_m is a predefined parameter (set to N_n in this section), resulting in a total of $N_n \times N_m$ potentially repeated states. These states can then be viewed as a probability distribution over all the states on the attractor by counting their occurrences and normalizing by $N_n \times N_m$, resulting in $p_{R_{t_\zeta}}$, as shown in Fig. S5. If the states in R_{t_ζ} are closely located in the phase space, the distribution will be more concentrated; conversely, if they are more spread, the distribution will be less concentrated. The same steps and analysis also apply to $R^\eta_{t_\zeta}$. If we now take the Shannon entropy perspective to characterize the predictability of a state ζ, we can compute the rate of change of the entropy over a time interval η: $\Delta H_\eta(\zeta) = H(p_{R_{t_\zeta}}) - H(p_{R^\eta_{t_\zeta}})$ (step 3, in Fig. S5). This quantity measures the expected amount of information created. The more information is created, the more unexpected the configuration, and thus the less predictable the original state. We note that, in the illustrative example presented in Fig. S5, $\Delta H_\eta(\zeta) > 0$, suggesting that $R^\eta_{t_\zeta}$ is more spread in the phase space than R_{t_ζ}.

We consider this an effective link between α_η and information theory, noting that it would require further in-depth analysis, which lies beyond the scope of this paper. Yet, we believe that the proposed methodology may pave the way for new applications of information theory in high-dimensional dynamical systems.

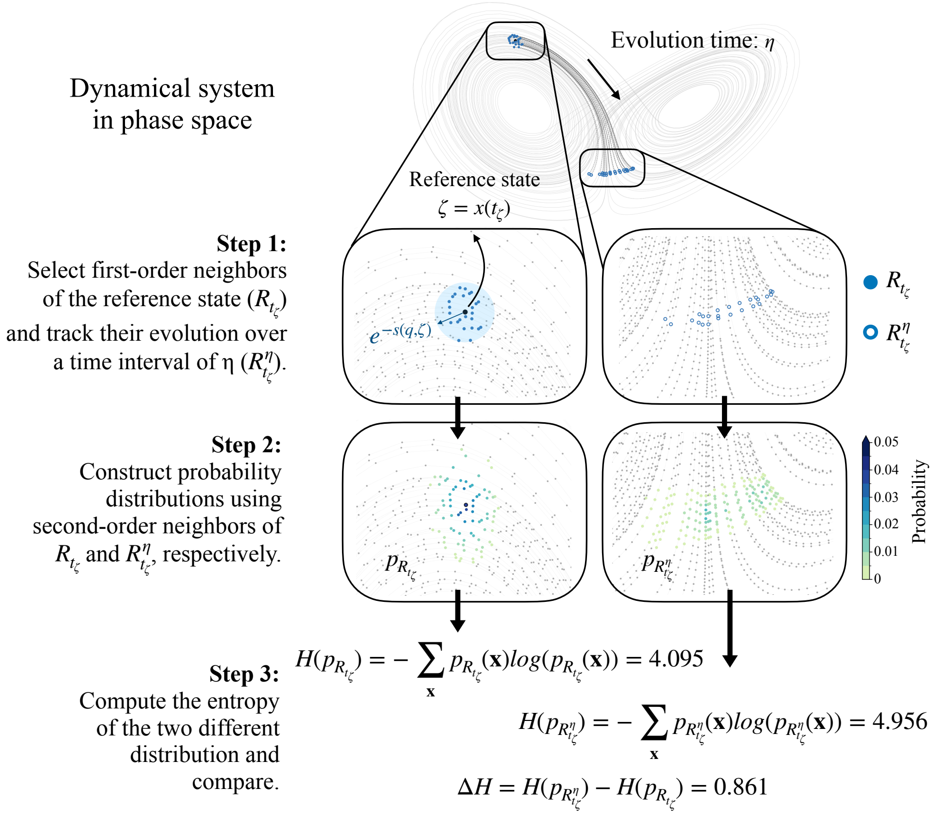

Fig. S5. Schematic illustration of the computation of entropy change $\Delta H_\eta(\zeta)$, demonstrated in the phase space of the Lorenz-63 system. The panels in the second row (step 1) provide a zoomed-in view of the phase space region where $\alpha_\eta(\zeta)$ is defined. *Recurrences* (R_{t_ζ}) are represented by solid blue dots, while *forward recurrences* ($R^\eta_{t_\zeta}$) are depicted by empty blue dots, as in Fig. 1. The blue circle with radius $e^{-s(q,\zeta)}$ indicates the hypersphere used to define the neighborhood of the reference state. In step 2, second-order neighbors of each state within R_{t_ζ} and $R^\eta_{t_\zeta}$ are taken to construct probability distributions $p_{R_{t_\zeta}}$ and $p_{R^\eta_{t_\zeta}}$, respectively. The panels in the third row are colored according to these distributions. In step 3, the entropy of these two distributions is computed and compared.

4. Comparison with Nonlinear Local Lyapunov Exponent

The Nonlinear Local Lyapunov Exponent (NLLE) was first proposed by Ding and Li in 2007 (5). Unlike previous approaches that rely on linearized dynamics, NLLE uses the full dynamics to characterize the error growth rate without linearizing the governing equations. When the governing equations of the dynamical system is not explicitly known, they provided a method for estimating NLLE from observational data based on analogous, as shown in (6). This index is sometimes also called the 'Local Growth Rate', with an open-source implementation provided in (7). In this section, we computed NLLE using the same Lorenz-63 dataset as used in the main analysis. Figure S6 demonstrates the distribution of NLLE (λ_η) for different η.

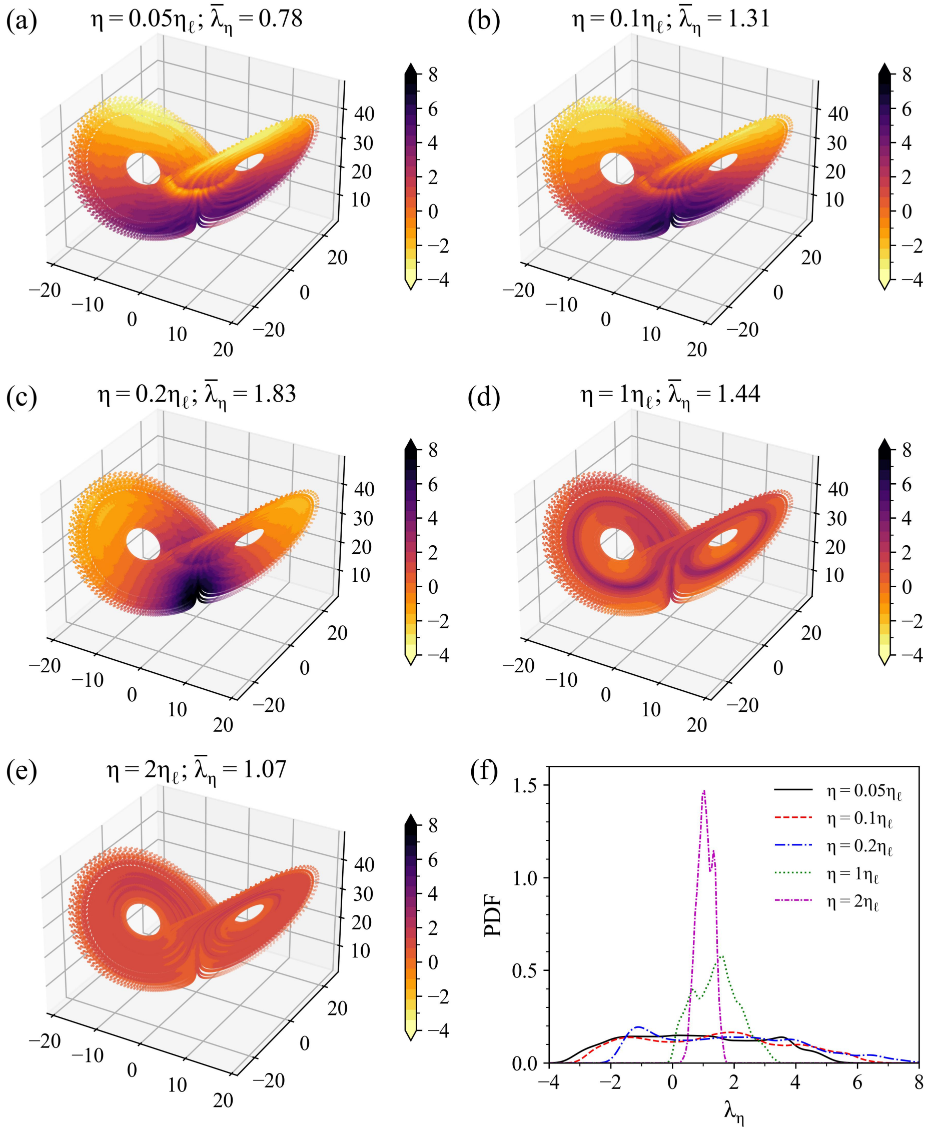

Fig. S6. Distribution of NLLE at different prediction horizons for the stochastic Lorenz-63 system. Same as Figure 2, but for the Nonlinear Local Lyapunov Exponent.

5. Extension of the Predictability Index

In this paper, we introduce a new method that characterizes the local predictability of a dynamical system by applying the concept that measuring predictability can be viewed as assessing the divergence rate of nearby trajectories. This is implemented by counting the number of trajectories that evolve closely with the reference trajectory, normalized to a bounded value using the size of the neighborhood. Despite this approach demonstrating potential in analyzing local predictability, it discards information about trajectories that have left the neighborhood, whose dynamics also reflect predictability to some extent. To this end, we proposed an extension of this predictability index by weighting all the nearby trajectories by their distances to the reference trajectory.

Recall that when deriving α_η for a given reference state ζ and forecasting horizon η, we defined three groups of states: *recurrences* (R_{t_ζ}), *forward recurrences* ($R^\eta_{t_\zeta}$) and *forward-reference-state recurrences* ($R_{t_\zeta+\eta}$). Based on these three groups of states, we define the value of the original version α_η as follows:

$$\alpha_\eta(\zeta) = \frac{|R^\eta_{t_\zeta} \cap R_{t_\zeta+\eta}|}{|R_{t_\zeta}|} \qquad [7]$$

To extend this index, we first track the distances of all states belonging to *forward recurrences* ($R^\eta_{t_\zeta}$) to the reference trajectory as D:

$$D = \left\{ \text{dist}\left(x(t), x(t_\zeta + \eta)\right) \quad \forall x(t) \in R^\eta_{t_\zeta} \right\}. \qquad [8]$$

where the dist function can be any distance metric, but we only consider L2 norm in this study.

Next, we also record the distances of states that belong to the intersection of *forward recurrences* ($R^\eta_{t_\zeta}$) and *forward-reference-state recurrences* ($R_{t_\zeta+\eta}$) to the reference trajectory. We denote these distances as D':

$$D' = \left\{ \text{dist}\left(x(t), x(t_\zeta + \eta)\right) \quad \forall x(t) \in R^\eta_{t_\zeta} \cap R_{t_\zeta+\eta} \right\}. \qquad [9]$$

Based on these distances, we further define the extension of the index $\alpha^*_\eta(\zeta)$ as:

$$\alpha^*_\eta(\zeta) = \frac{\sum_{d' \in D'} \phi(d')}{\sum_{d \in D} \phi(d)} \qquad [10]$$

where ϕ can be a custom positive function used to make the extension focus on different aspects, as further explained later. Since D' is a subset of D, the values of α^*_η, like α_η, are also defined to lie within the range of 0 to 1. Specifically, we note that the original α_η can be viewed as a special case of the extended index α^*_η when $\phi(x) = x^0$, meaning that all trajectories are not weighted by their distances to the reference trajectory.

In Figure S7 and S8, we show the results of α^*_η using $\phi(x) = x^{-1}$ and $\phi(x) = x$, respectively. For these two different $\phi(x)$, the results are generally similar to those obtained when $\phi(x) = 1$, although they show noticeable differences in the details. When $\phi(x) = x^{-1}$, α^*_η decreases slowly as the forecasting horizon increases, whereas the opposite holds for $\phi(x) = x^1$. This can be explained as $\phi(x) = x^{-1}$ assigns higher weights to the close neighbors, while $\phi(x) = x^1$ assigns higher weights to the states that are farther away. This means we can selectively define ϕ based on the needs of different tasks. For instance, in some cases, we may be particularly interested in fast diverging trajectories, making $\phi(x) = x^1$ a more suitable choice.

To sum up, this extension uses distances as weights, incorporating more information compared to the original α_η which relies on binary values. It also estimates local predictability, and with the extensible nature provided by the weighting function ϕ, it offers additional information for analysis.

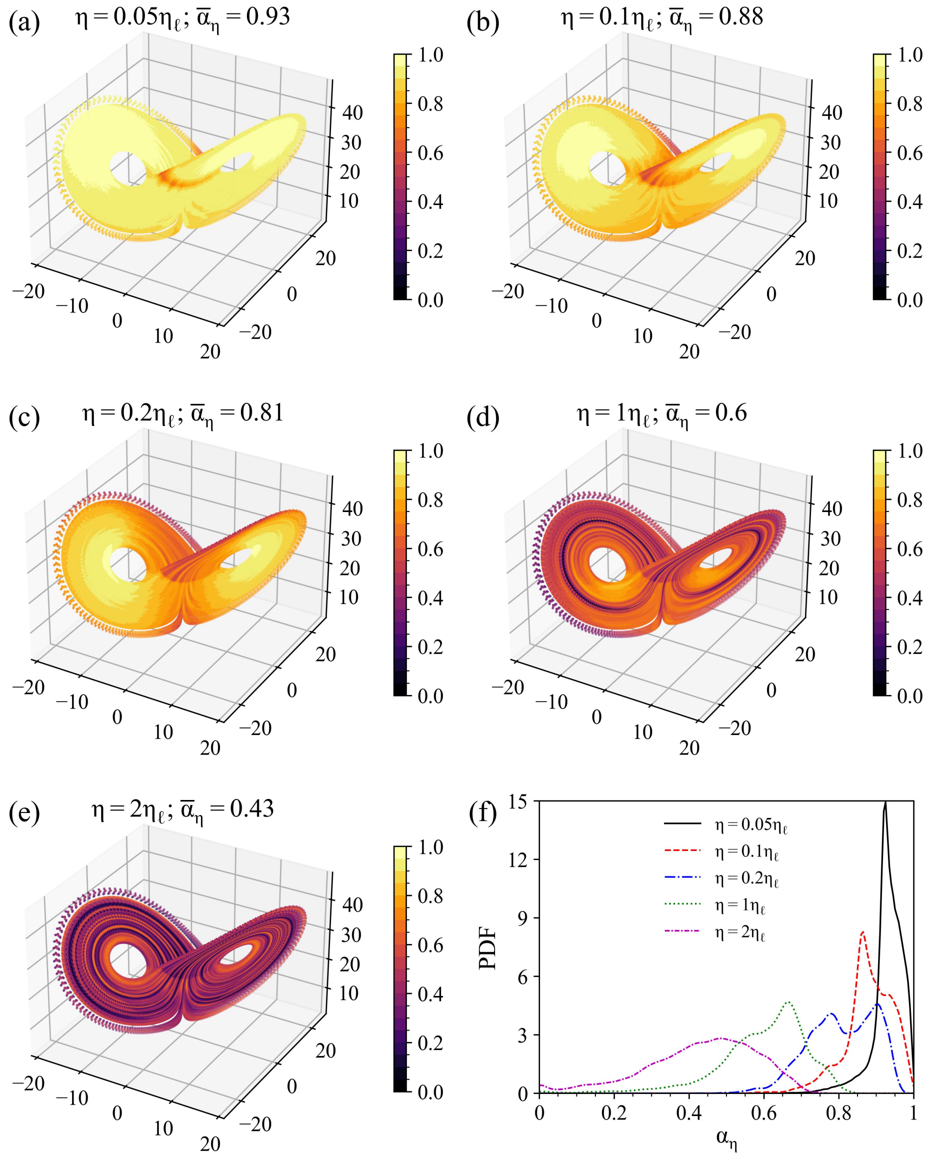

Fig. S7. Distribution of α_η^* at different prediction horizons for the Lorenz-63 system, with $\phi(x) = x^{-1}$. Same as Figure 2, but for the extension of the predictability index.

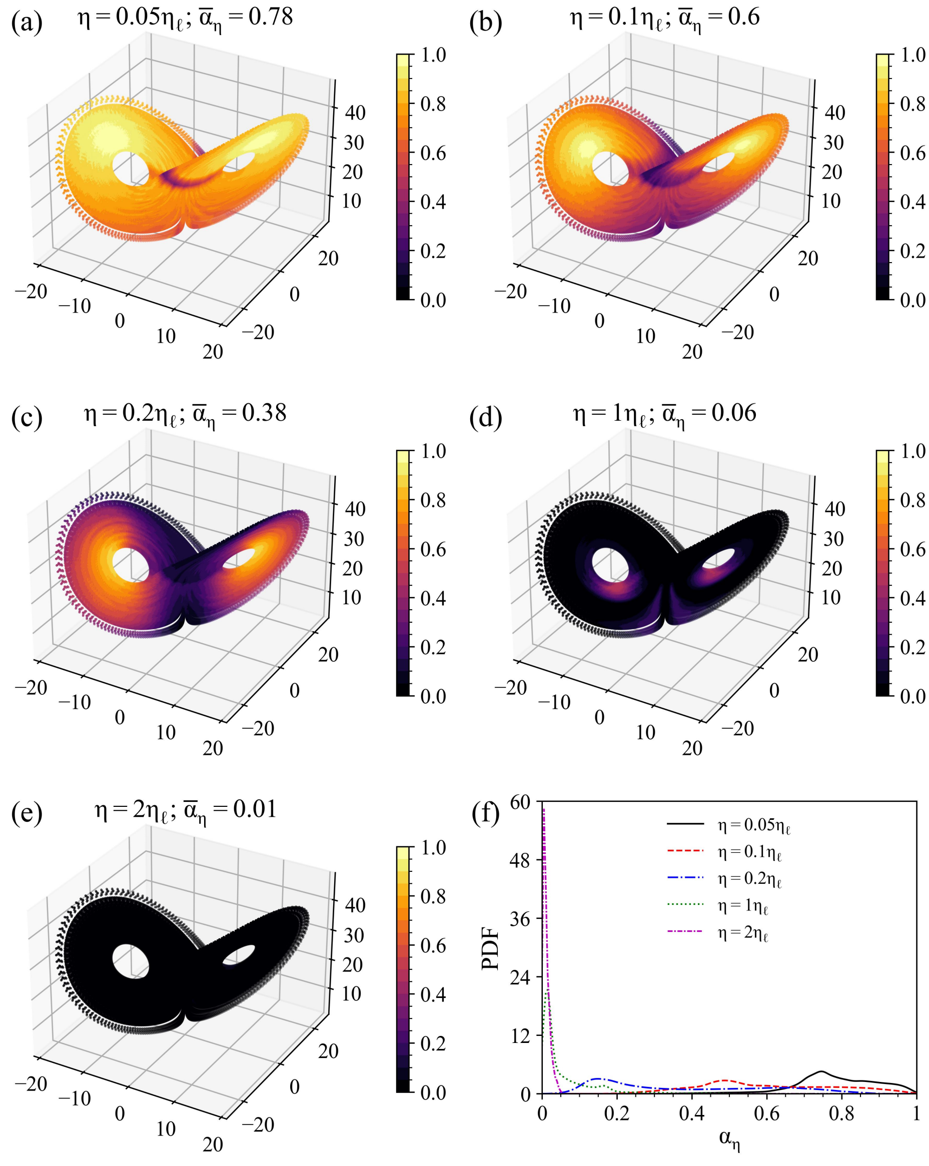

Fig. S8. Distribution of α_η^* at different prediction horizons for the Lorenz-63 system, with $\phi(x) = x$. Same as Figure 2, but for the extension of the predictability index.

6. Definition of North Atlantic Wintertime Weather Regimes

The definition of weather regimes is adapted from (8), focusing on the wintertime (DJFM) 500 hPa (Z500) in the Euro-Atlantic sector (80°W to 50°E, 22.5°N to 70°N). The weighted Z500 daily anomaly data is decomposed using EOF analysis, retaining the 14 leading modes (accounting for approximately 90% of the variance) to speed up the computation. The climatology is computed as the calendar day mean field of Z500 from 1979 to 2022, based on a centered 15-day window, while the weight is applied to the data as a cosine function of the latitude. The k-means clustering algorithm is then applied to the principal components, resulting in four weather regimes, whose centroids are demonstrated in Fig. S9. Next, we compute the normalized projection of each daily Z500 anomaly field onto the cluster centroid, which has been termed the Weather Regimes Index (IWR) in previous studies (9). Here, we aim to analyze the predictability of established weather regimes; therefore, we require that the Weather Regimes Index (IWR) remains above 1.0 for at least 5 days to classify a state as belonging to a weather regime. States that are not allocated to any weather regime are classified as "no regime".

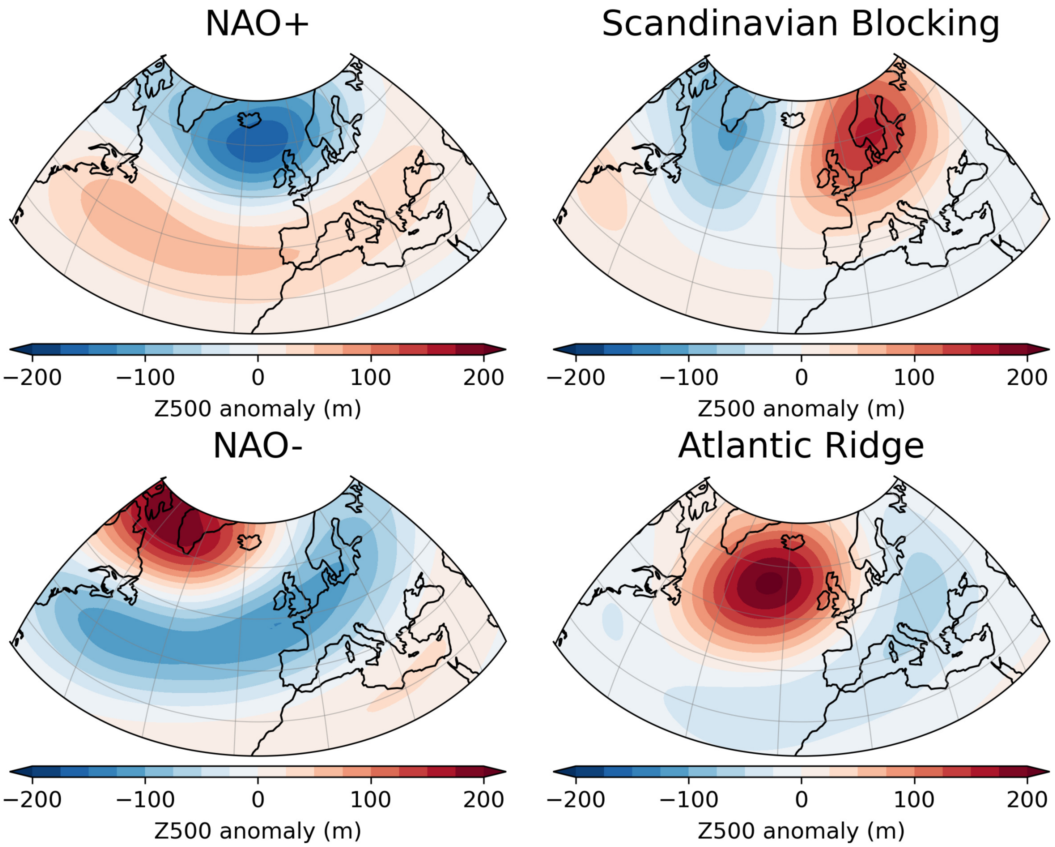

Fig. S9. Composite Z500 anomaly maps for the four weather regimes in the North Atlantic-Europe region.

7. Datesets for other examples

A. Rössler system. The Rössler system is a set of three nonlinear ordinary differential equations originally introduced in 1976 (10). Its governing equations can be written as:

$$\frac{dx}{dt} = -y - z, \quad [11]$$

$$\frac{dy}{dt} = x + ay, \quad [12]$$

$$\frac{dz}{dt} = b + z(x - c). \quad [13]$$

In this study, we adopt the classical parameters of the Rössler system that exhibit deterministic chaos, where $(a, b, c) = (0.2, 0.2, 5.7)$. Observational data are obtained through numerical simulation with a temporal resolution of $dt = 0.005$ over 10^5 time steps.

B. Spring-slider system for laboratory earthquakes. In (11), one spring-slider model is developed to simulate the dynamics of slow earthquakes. This model is described by the following set of ordinary differential equations (ODEs):

$$\dot{x} = \frac{e^x\left[(\beta_1 - 1)\,x(1+\lambda u) + y - u\right] + \kappa\left(\frac{v_0}{v_*} - e^x\right) - \dot{u}\frac{1+\lambda y}{1+\lambda u}}{1 + \lambda u + v e^x} \quad [14]$$

$$\dot{y} = \kappa\left(\frac{v_0}{v_*} - e^x\right) - v e^x \dot{x} \quad [15]$$

$$\dot{z} = -\rho e^x\left(\beta_2 x + z\right) \quad [16]$$

$$\dot{u} = -\alpha - \gamma u + \dot{z}. \quad [17]$$

where the state of system can be fully characterized by the state vector $[x, y, z, u]$. To further simulate the irregularity of labquakes, two stochastic terms modeled by a Wiener process are added to the equations for y and u, making the system a set of Stochastic Differential Equations (SDE):

$$\mathrm{d}y = \left[\kappa\left(\frac{v_0}{v_*} - e^x\right) - v e^x \dot{x}\right]\mathrm{d}T + \varepsilon_y\,\mathrm{d}W_T \quad [18]$$

$$\mathrm{d}u = [-\alpha - \gamma u + \dot{z}]\mathrm{d}T + \varepsilon_u\,\mathrm{d}W_T \quad [19]$$

The values of all the parameters are presented in Table S1, while their physical explanations are available in (11). We generated $2 * 10^5$ time steps with $dt = 0.01$ for the analysis. In Figure 7, the attractor for the spring-slider system is visualized only only using variables x, y, and z, given the limitation to three dimensions.

Table S1. Simulation Parameters

Parameter	Value
β_1	1.2
β_2	0.8265
λ	0.0156
κ	0.2555
v_0	10^{-6}
v_*	10^{-6}
v	0.004
ρ	0.1
α	0.0112
γ	0.03
ε_y	0.0115
ε_u	0.0111

C. Double Pendulum, EEG and ECG. The data of these three systems are from (12), with the double pendulum data generated through numerical simulation, while the other two are obtained from observational biological data. The EEG and ECG data were adapted from the PhysioNet database (13–15).

For the double pendulum, the simulations were conducted using a variational integrator based on the Euler-Lagrange equations:

$$\frac{\mathrm{d}}{\mathrm{d}t}\frac{\partial L}{\partial \dot{\mathbf{q}}} - \frac{\partial L}{\partial \mathbf{q}} = 0, \quad [20]$$

with the Lagrangian $L = T - V$ is the kinetic (T) minus potential (V) energy. For double pendulum, $\mathbf{q} = [\theta_1 \quad \theta_2]^T$, and the Lagrangian (L) becomes:

$$L = \frac{1}{2}(m_1 + m_2)l_1^2\dot{\theta}_1^2 + \frac{1}{2}m_2 l_2^2\dot{\theta}_2^2 + m_2 l_1 l_2 \dot{\theta}_1\dot{\theta}_2 \cos(\theta_1 - \theta_2) - (m_1 + m_2)l_1 g(1 - \cos\theta_1) - m_2 l_2 g(1 - \cos\theta_2). \qquad [21]$$

where $l_1 = l_2 = m_1 = m_2 = 1$, $g = 10$. The action integral was approximated using the trapezoidal rule to compute the equations of motion:

$$\delta \int_a^b L(\mathbf{q}, \dot{\mathbf{q}}, t)\,\mathrm{d}t = 0. \qquad [22]$$

$x(t) = \sin(\theta_1)$ is used as the observational time series for double pendulum.

For all these three examples, a similar approach to (12) is applied to the time series data to obtain eigen-time-delay coordinates. Specifically, a time series x(t) is stacked into a Hankel matrix H, which is then decomposed using singular value decomposition (SVD):

$$\mathbf{H} = \begin{bmatrix} x(t_1) & x(t_2) & \cdots & x(t_p) \\ x(t_2) & x(t_3) & \cdots & x(t_{p+1}) \\ \vdots & \vdots & \ddots & \vdots \\ x(t_q) & x(t_{q+1}) & \cdots & x(t_m) \end{bmatrix} = \mathbf{U}\Sigma\mathbf{V}^*. \qquad [23]$$

This yields a $\mathbf{V}^*$ matrix, which represents a hierarchy of eigen time series that reconstruct a delay-embedded attractor. The predictability index α_η is computed based on the $\mathbf{V}^*$ matrix and visualized on the attractor constructed from the three leading time series in $\mathbf{V}^*$ (see Fig. 7). A summary of the parameters used for the construction and decomposition of the Hankel matrix is provided in Table S2. We note that the parameters used here differ from those in (12) to retain higher energy for predictability analysis.

Table S2. Summary of Parameters

System	Type	Measured Variable	Time Step Δt	# Rows in H q	Rank r	Energy in r Modes (%)
Double Pendulum	ODE	$x(t)$	0.001	100	5	99.9
ECG	Data	Voltage	0.004s	25	5	93.3
EEG	Data	Voltage	0.01s	200	8	61.2

8. Real-time Predictability Analysis

In certain scenarios, such as weather forecasting, real-time predictability analysis is important for evaluating and informing the operational forecast system. However, the computation of α_η rely on the future trajectory of the reference state ζ', therefore hindering direct real-time applications. As shown in Equation 9 in the main text, we propose a proxy that do not rely on the future information of the reference state. The idea of this proxy stems from the fact that the predictability index represents local predictability in the phase space. Therefore, we can use the average predictability of the neighboring states of ζ' to represent its predictability.

An example of a real-time application is presented using the Lorenz-63 system, following the same numerical simulation scheme as other experiments in this study. We assume that we have 10^5 time steps as historical observations, and their corresponding α_η have already been computed, as shown in Fig. S10 (b,d,f). Meanwhile, we also assume there are 2000 states not present in the historical observations, which are used to simulate real-time unseen states in an operational forecast system. Figure S10 (a,c,e) shows the predictability proxy for the unseen states. By comparing the results with the reference α_η, we note that the proposed proxy can effectively capture the features of predictability, regardless of the region in phase space or the forecasting horizon. Specifically, the low predictability near the lobe transition region is in Fig. S10 (a,c), and the high predictability region near the fixed point in Fig. S10 (e) are both well reflected. Although this application is a rather simple case, we anticipate its application in other operational forecast systems.

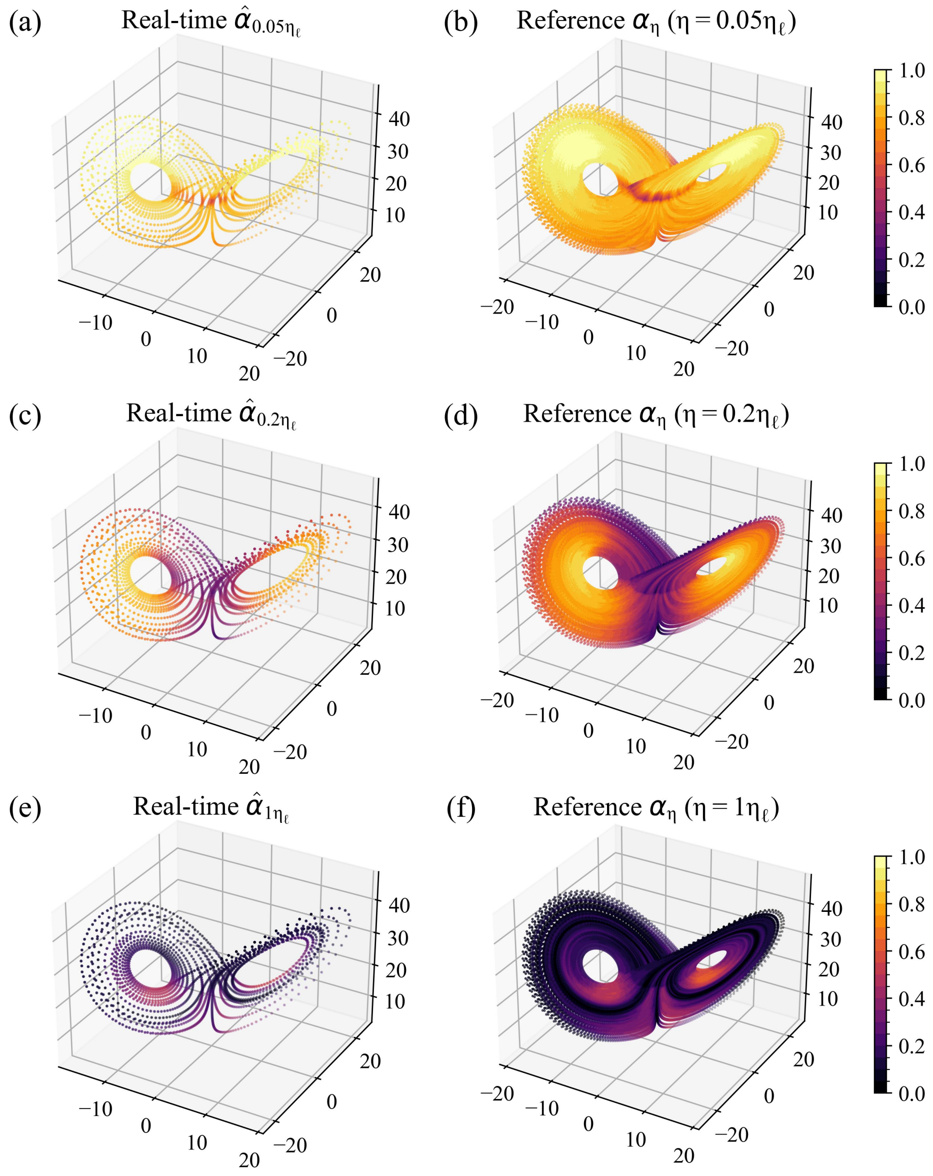

Fig. S10. Real-time predictability analyses for the Lorenz system. (a,c,e) 2000 unseen Lorenz-63 states colored according to the predictability index proxy $\hat{\alpha_\eta}$, presented across three different forecasting horizons: 0.05η_ℓ (a), 0.2η_ℓ (c), and 1η_ℓ (e). Panels (b), (d), and (f) display the reference α_η, as in 2 (a), (c), and (d).

References

1. EN Lorenz, Deterministic nonperiodic flow. *J. atmospheric sciences* **20**, 130–141 (1963).
2. D Faranda, G Messori, P Yiou, Dynamical proxies of north atlantic predictability and extremes. *Sci. reports* **7**, 41278 (2017).
3. G Boffetta, P Giuliani, G Paladin, A Vulpiani, An extension of the lyapunov analysis for the predictability problem. *J. Atmospheric Sci.* **55**, 3409–3416 (1998).
4. E Aurell, G Boffetta, A Crisanti, G Paladin, A Vulpiani, Predictability in the large: an extension of the concept of lyapunov exponent. *J. physics A: Math. general* **30**, 1 (1997).
5. R Ding, J Li, Nonlinear finite-time lyapunov exponent and predictability. *Phys. Lett. A* **364**, 396–400 (2007).
6. J Li, R Ding, Temporal–spatial distribution of atmospheric predictability limit by local dynamical analogs. *Mon. Weather. Rev.* **139**, 3265–3283 (2011).
7. G Datseris, U Parlitz, *Nonlinear dynamics: a concise introduction interlaced with code.* (Springer Nature), (2022).
8. C Cassou, Intraseasonal interaction between the madden–julian oscillation and the north atlantic oscillation. *Nature* **455**, 523–527 (2008).
9. C Michel, G Rivière, The link between rossby wave breakings and weather regime transitions. *J. Atmospheric Sci.* **68**, 1730–1748 (2011).
10. OE Rössler, An equation for continuous chaos. *Phys. Lett. A* **57**, 397–398 (1976).
11. A Gualandi, D Faranda, C Marone, M Cocco, G Mengaldo, Deterministic and stochastic chaos characterize laboratory earthquakes. *Earth Planet. Sci. Lett.* **604**, 117995 (2023).
12. SL Brunton, BW Brunton, JL Proctor, E Kaiser, JN Kutz, Chaos as an intermittently forced linear system. *Nat. communications* **8**, 19 (2017).
13. AL Goldberger, et al., Physiobank, physiotoolkit, and physionet: components of a new research resource for complex physiologic signals. *circulation* **101**, e215–e220 (2000).
14. P Laguna, RG Mark, A Goldberg, GB Moody, A database for evaluation of algorithms for measurement of qt and other waveform intervals in the ecg in *Computers in cardiology 1997.* (IEEE), pp. 673–676 (1997).
15. B Kemp, AH Zwinderman, B Tuk, HA Kamphuisen, JJ Oberye, Analysis of a sleep-dependent neuronal feedback loop: the slow-wave microcontinuity of the eeg. *IEEE Transactions on Biomed. Eng.* **47**, 1185–1194 (2000).
